# Structural Investigations of the Inhibition of Escherichia coli AmpC β-Lactamase by Diazabicyclooctanes

**DOI:** 10.1128/AAC.02073-20

**Published:** 2021-01-20

**Authors:** Pauline A. Lang, Thomas M. Leissing, Malcolm G. P. Page, Christopher J. Schofield, Jürgen Brem

**Affiliations:** a Department of Chemistry, Chemistry Research Laboratory, University of Oxford, Oxford, United Kingdom; b Jacobs University Bremen gGmbH, Bremen, Germany

**Keywords:** antimicrobial resistance, serine β-lactamase inhibitors, diazabicyclooctane, avibactam, relebactam, nacubactam, zidebactam, Avycaz, cephalosporin resistance

## Abstract

β-Lactam antibiotics are presently the most important treatments for infections by pathogenic Escherichia coli, but their use is increasingly compromised by β-lactamases, including the chromosomally encoded class C AmpC serine-β-lactamases (SBLs). The diazabicyclooctane (DBO) avibactam is a potent AmpC inhibitor; the clinical success of avibactam combined with ceftazidime has stimulated efforts to optimize the DBO core. We report kinetic and structural studies, including four high-resolution crystal structures, concerning inhibition of the AmpC serine-β-lactamase from E. coli (AmpC*_EC_*) by clinically relevant DBO-based inhibitors: avibactam, relebactam, nacubactam, and zidebactam. Kinetic analyses and mass spectrometry-based assays were used to study their mechanisms of AmpC*_EC_* inhibition. The results reveal that, under our assay conditions, zidebactam manifests increased potency (apparent inhibition constant [*K*_iapp_], 0.69 μM) against AmpC*_EC_* compared to that of the other DBOs (*K*_iapp_ = 5.0 to 7.4 μM) due to an ∼10-fold accelerated carbamoylation rate. However, zidebactam also has an accelerated off-rate, and with sufficient preincubation time, all the DBOs manifest similar potencies. Crystallographic analyses indicate a greater conformational freedom of the AmpC*_EC_*-zidebactam carbamoyl complex compared to those for the other DBOs. The results suggest the carbamoyl complex lifetime should be a consideration in development of DBO-based SBL inhibitors for the clinically important class C SBLs.

## Abstract (precis)

β-Lactam antibiotics are presently the most important treatments for infections by pathogenic Escherichia coli, but their use is increasingly compromised by β-lactamases, including the chromosomally encoded class C AmpC serine-β-lactamases (SBLs). The diazabicyclooctane (DBO) avibactam is a potent AmpC inhibitor; the clinical success of avibactam combined with ceftazidime has stimulated efforts to optimize the DBO core.

## INTRODUCTION

**FIG 1:**
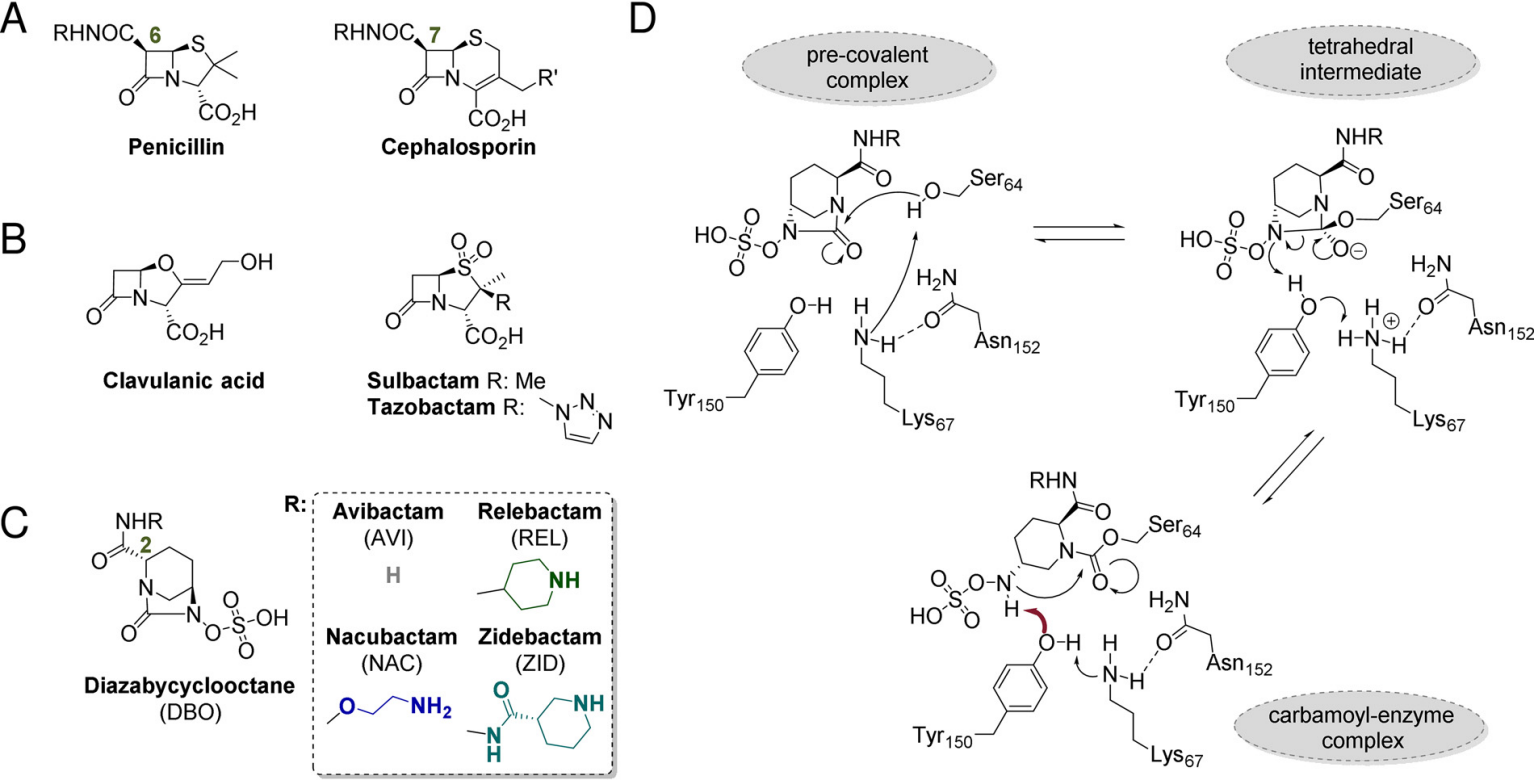
β-Lactam antibiotics and β-lactamase inhibitors. (A) Penicillin and cephalosporin antibiotics. (B) Established β-lactam-based SBL inhibitors. (C) Diazabicyclooctane (DBO)-based SBL inhibitors with different C2 acyl amido sidechains. The different colors for the inhibitor sidechains are used throughout. (D) Proposed mechanisms for binding and recyclization of DBOs to class C SBLs (29). While either a two-base mechanism, including K67 and Y150 (shown here), or a single-base mechanism involving only K67 (not shown) has been proposed for deprotonation and reprotonation of S64, in both scenarios recyclization requires deprotonation of the DBO *N*-sulfate nitrogen by Y150 (29).

Infections by pathogenic Escherichia coli are a major worldwide health concern (1–3) and are often treated with β-lactam antibiotics (e.g., penicillins and cephalosporins) (Fig. 1A). However, β-lactamase-mediated resistance to these and other β-lactams is increasing (4). There are two structural/mechanistic groups of β-lactamases, the nucleophilic serine-β-lactamases (SBLs; Ambler classes A, C, and D) and the zinc-dependent metallo-β-lactamases (MBLs; Ambler class B), of which the SBLs are presently more abundant and, therefore, of particular clinical concern (5). While no MBL inhibitors have been approved for clinical use, established SBL inhibitors (clavulanic acid [6], sulbactam [7], and tazobactam [8] [Fig. 1B]) are potent inhibitors of many class A SBLs (excepting carbapenemases), although their inhibition of class C and class D SBLs is much weaker (9). The AmpC type class C SBLs, unlike many β-lactamases, are chromosomally encoded and present in most E. coli strains (10). Mutations in promoter and attenuator regions lead to hyperproduction of AmpC in pathogenic E. coli (11, 12), conferring resistance to first-, second-, and third-generation cephalosporins and most penicillins, although as yet not to carbapenems (10).

The clinical introduction of ceftazidime-avibactam (CZA; Avycaz) was a milestone in treatment of infections caused by bacteria producing class C SBLs, including AmpC SBLs (13). The diazabicyclooctane (DBO) (Fig. 1A) avibactam (AVI; formerly NXL-104) is the first clinically useful non-β-lactam β-lactamase inhibitor and has potent activity against many class A and C and some class D SBLs (14).

Contrary to the proposed mechanisms of the established β-lactam SBL inhibitors, which involve essentially irreversible formation and subsequent hydrolysis of acyl-enzyme complexes, the cyclic urea carbonyl of avibactam reacts reversibly with the nucleophilic serine residue to form a carbamoyl-enzyme complex (Fig. 1D) (14–16). The success of avibactam has promoted efforts to optimize DBO-containing SBL inhibitors, in particular by modification of the C2 amido sidechain, the binding of which approximately mimics the respective C6 or C7 amido sidechains of the penicillins and cephalosporins (Fig. 1A). These have yielded several promising new inhibitors, including relebactam (REL), nacubactam (NAC), and zidebactam (ZID) (17–21). While the interactions of avibactam with a wide variety of SBLs have been studied (e.g., see references 22–28), structural information on the binding of other DBOs is more limited (17, 18, 29–32).

To enable structure-activity relationship (SAR)-guided efforts on DBOs, we now report kinetic and crystallographic studies on the inhibition of the AmpC SBL from Escherichia coli (AmpC*_EC_*) by avibactam, relebactam (20) (formerly MK-7655, used in combination with imipenem and cilastatin [Recarbrio]), nacubactam (18) (formerly OP-0595, currently in phase II), and zidebactam (19) (formerly WCK-5107, currently in phase II). The results reveal a distinctive kinetic profile for AmpC*_EC_* inhibition by ZID, which differs from those observed for AVI, REL, and NAC. Together with studies on the inhibition of class A, D, and other structurally distinct class C SBLs (17, 18, 29–32), the results will help guide further optimization of the DBO scaffold for potent broad-spectrum SBL inhibition.

## RESULTS AND DISCUSSION

### Zidebactam shows enhanced activity against isolated AmpC*_EC_* compared to other DBOs due to rapid binding.

Steady-state competitive inhibition kinetics of AmpC*_EC_* by AVI, REL, NAC, and ZID were measured using the fluorescent substrate FC-5 (33) and analyzed as previously described (34). All the tested DBOs were potent inhibitors for AmpC*_EC_*, with apparent inhibition constant (*K*_iapp_) values ranging from 7.4 μM for AVI to 0.69 μM for ZID. Without preincubation of the DBO inhibitor with AmpC*_EC_*, ZID is ∼10-fold more potent (pIC_50_ [negative log of IC_50_], 7.60) than AVI, REL, or NAC (pIC_50_s of 6.62, 6.61, and 6.75, respectively). However, with prolonged incubation (10 to 360 min), all DBOs exhibited similar potencies (pIC_50_s of 8.16, 8.41, 8.53, and 8.46, respectively; see Table 1 and see Table S3 and Fig. S3D in the supplemental material). Assays at pH 7.0 to 8.0 did not show a significant pH dependence of inhibition (Table S3).

**TABLE 1:** Kinetic analysis of AmpC*_EC_* inhibition by DBOs^*a*^

DBO	*K*_iapp_ (μM)	*k*_2_/*K* (M^−1^ s^−1^), ×10^3^	*k*_off_ (s^−1^), ×10^−3^	*t*_1/2_ (min)	pIC_50_	
					0 min	360 min
AVI	7.4 ± 0.3	36 ± 1	0.060 ± 0.01	192 ± 1	6.62 ± 0.04	8.16 ± 0.02
REL	7.1 ± 0.3	38 ± 1	0.032 ± 0.01	364 ± 2	6.61 ± 0.04	8.41 ± 0.01
NAC	5.0 ± 0.1	52 ± 2	0.049 ± 0.01	236 ± 1	6.75 ± 0.04	8.53 ± 0.02
ZID	0.69 ± 0.04	360 ± 10	0.350 ± 0.01	39 ± 4	7.60 ± 0.02	8.46 ± 0.02

a *K*_iapp_ values and pseudo first-order rates (*k*_2_/*K*) were determined by assaying 100 nM AmpC*_EC_* with 5 μM FC-5 (33). *k*_off_ rates were determined by jump dilution (100,000-fold) of AmpC*_EC_* (1 μM) that had been preincubated with AVI, REL, NAC, or ZID (10 μM) at room temperature for 30 min and then assayed using 25 μM FC-5. pIC_50_s were obtained from assays using 500 pM AmpC*_EC_* and 5 μM FC-5 with the respective preincubation time at room temperature. The buffer was 50 mM Tris, pH 7.5, 0.01% (vol/vol) Triton X-100. Data were analyzed as described in Materials and Methods.

More detailed studies showed that carbamoylation of AmpC*_EC_* by ZID is ∼10-fold faster than that for the other tested DBOs (360 × 10^3^ M^−1^ s^−1^ compared to 36 × 10^3^ M^−1^ s^−1^, 38 × 10^3^ M^−1^ s^−1^, and 52 × 10^3^ M^−1^ s^−1^ for AVI, REL, and NAC, respectively), rationalizing its increased potency under the assay conditions without preincubation. However, the decarbamoylation rate of ZID, as measured by the jump-dilution method, is also accelerated (0.35 × 10^−3^ s^−1^ compared to 0.060 × 10^−3^ s^−1^, 0.032 × 10^−3^ s^−1^, and 0.049 × 10^−3^ s^−1^ for AVI, REL, and NAC, respectively) (Table 1).

Comparing the steady-state kinetics of AmpC*_EC_* inhibition by AVI, REL, and ZID to reported results for the Acinetobacter baumannii-derived class C cephalosporinase ADC-7 and the Pseudomonas aeruginosa-derived class C cephalosporinase PDC-3 reveals similar trends in potency, i.e., similar differences in carbamoylation as well as decarbamoylation rates are clear for the four different DBOs studied here (Table S4) (17). Thus, relatively fast binding likely contributes to the generally improved potency of ZID compared to AVI, REL, and NAC versus class C SBLs, at least with short incubation times.

### DBOs restore CAZ activity against AmpC-expressing E. coli.

To confirm AmpC*_EC_* inhibition by the DBOs in cells, they were tested against DH5α Escherichia coli and the same strain carrying the pAD7 AmpC*_EC_* plasmid (35). First, the intrinsic antibacterial activity of DBOs was confirmed, with MICs of 16 μg ml^−1^ for AVI, 128 μg ml^−1^ for REL, 2 μg ml^−1^ for NAC, and ≤0.25 μg ml^−1^ for ZID being observed, both against the wild-type DH5a and the AmpC*_EC_*-expressing DH5a (Table S5). This is in agreement with reported MIC values with E. coli ATCC 25922 (16 μg ml^−1^ for AVI, >64 μg ml^−1^ for REL, 2 μg ml^−1^ for NAC, and 0.125 μg ml^−1^ for ZID) (36). The observed antimicrobial activity of DBOs against E. coli has been attributed to different degrees of inhibition of penicillin binding protein 2 (PBP-2) (18, 37).

Second, the DBOs were then tested in combination with the cephalosporin CAZ. The AmpC*_EC_*-producing strain was significantly less susceptible to CAZ than the wild type (MIC of 256 μg ml^−1^ compared to 1 μg ml^−1^). All DBOs fully restored ceftazidime activity when tested at 4 μg ml^−1^ and in a dose-dependent manner below their MICs (Table S5). Ceftazidime is a known potent PBP-3 inhibitor (38), and synergistic effects of the DBOs PBP-2 inhibition have been reported (39).

### Protein-observed SPE-MS.

Protein-observed solid-phase extraction mass spectrometry (SPE-MS) assays were utilized to investigate the reversible character of the DBO binding. After incubation with 1.1 equivalents of DBOs, rapid reaction was observed in all cases, indicating complete AmpC*_EC_* carbamoylation within 1 min. DBO adducts with mass increments of +265 Da, +338 Da, +324 Da, or +391 Da were observed, consistent with covalent modification of AmpC*_EC_* by AVI, REL, NAC, and ZID, respectively (Fig. S4 and S6). In agreement with the accelerated carbamoylation on AmpC*_EC_* by ZID compared to the other DBOs, as implied by the kinetic assays, direct competition experiments involving addition of AmpC*_EC_* to a 1:1 mixture of AVI with either REL, NAC, or ZID after 1 min showed preferred initial binding of ZID over AVI (77:23), while REL and NAC showed almost equivalent binding compared with AVI (44:56 and 49:51, respectively).

**FIG 2:**
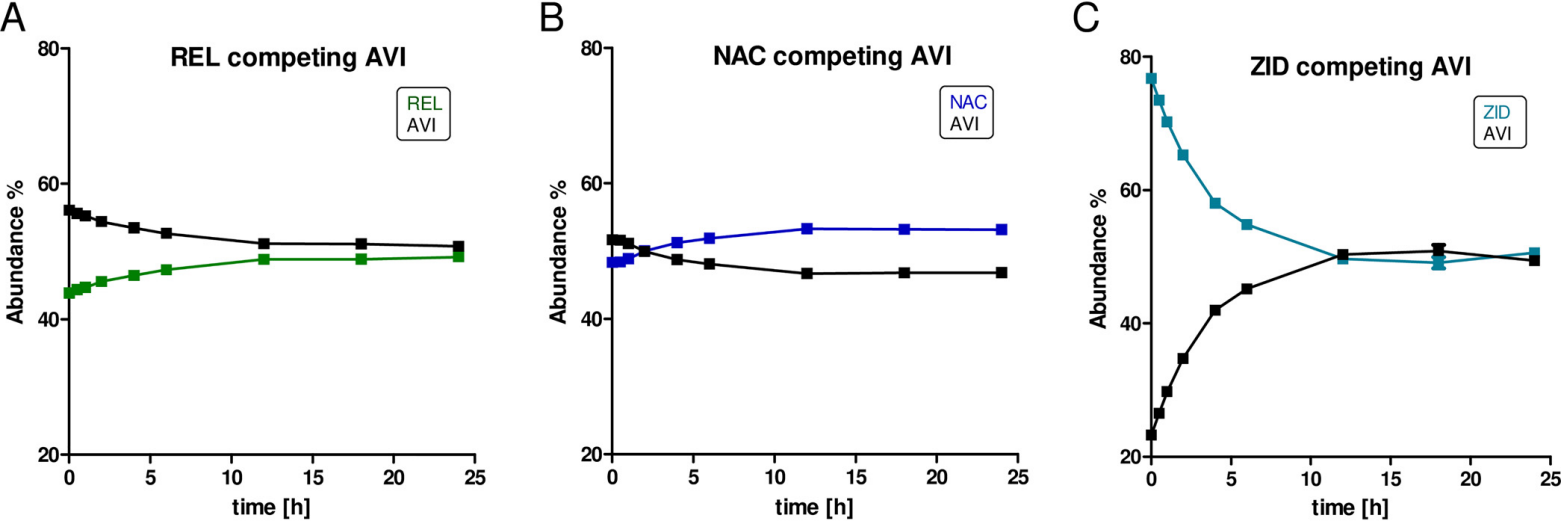
Time courses for DBO binding in competition with AVI measured by mass spectrometry. AmpC*_EC_* (3 μM) was added to a mixture of AVI (3.3 μM) and NAC, REL, or ZID (3.3 μM) in 50 mM Tris, pH 7.5. Experiments were performed in duplicate. Reactions were monitored using protein-observed SPE-MS. Spectra were deconvoluted (Fig. S4) using the maximum entropy algorithm in the MassHunter Workstation Qualitative Analysis V.7 program (Agilent Technologies). The relative peak areas of the +265-Da, +338-Da, +324-Da, or +391-Da adducts for AVI, REL, NAC, and ZID, respectively, are plotted. Low-abundancy signals with −80-Da mass increments relative to the intact DBO complexes were observed for all DBO carbamoyl-enzyme complexes but with various intensities for different DBOs (these are likely artifacts of the MS process [see the text and Fig. S6]); here, peak areas of the –80-Da species were added to those for the intact adducts.

Monitoring of the reactions over a 24-h period revealed equilibration of the DBO-derived AmpC*_EC_*-carbamoyl complexes. While REL and NAC were still bound in amounts similar to those of AVI (49:51 and 53:47, respectively) after 12 h, the ZID carbamoyl-enzyme complex showed a constant ratio of 51:49 (ZID:AVI) after 12 h of equilibration (Fig. 2 and Fig. S5). Studies where REL, NAC, or ZID were added to the AVI carbamoyl-enzyme complex, derived by preincubation of AmpC*_EC_* with AVI, showed equilibration to the same ratios of AVI to REL/NAC/ZID after 12 to 24 h (Fig. S5). Equilibration toward the same ratios of AVI to REL/NAC/ZID after 12 to 24 h were also observed when starting from the respective REL-, NAC-, and ZID-carbamoyl-enzyme complexes followed by addition of AVI (Fig. S5). Overall, these observations are in broad agreement with the inhibition studies, which show enhanced potency of ZID compared to AVI, REL, and NAC with short, but not with prolonged, incubation times.

A second mechanism, in addition to recyclization, for release of covalently linked DBOs from class A Klebsiella pneumoniae carbapenemase (KPC) β-lactamases has been reported; this involves the loss of the DBO *N*-sulfate group followed by hydrolysis of the resulting imine intermediate (e.g., see references 15 and 32). For AVI and NAC, small amounts of signals corresponding to the respective carbamoylated enzymes at −80 Da were observed, consistent with desulfation of the bound DBO. However, after 24 h the samples were reanalyzed, and no changes in the ratios of the intact −80-Da complexes were observed (Fig. S6). No evidence of further desulfation, or recovery of the unmodified enzyme, was observed over a period of 24 h (Fig. S6B). These observations contrast with observations for DBO fragmentation by KPC type SBLs, where the relative amounts of the −80-Da species compared to the intact complex were observed to increase with time (e.g., see references 15 and 32). Since the ratio of the intact inhibitor complex to the −80-Da complex remains consistent in our study, it is probable that in our observations the loss of sulfate is not a result of an enzyme reaction but is an artifact of the MS method, as reported previously (15).

### Structural basis of AmpC*_EC_* inhibition by DBOs.

As both carbamoylation and decarbamoylation rates of ZID for AmpC*_EC_* are accelerated, its unusual kinetic profile compared to the other tested DBOs at least in part may result from a reduced activation energy resulting from better stabilization of the common high-energy tetrahedral intermediate involved in carbamoylation and decarbamoylation (16) (Fig. 1D) rather than interactions that stabilize the carbamoyl-enzyme complex of AmpC*_EC_* (40, 41). To investigate the basis for the kinetics observed for ZID compared to other DBOs against AmpC*_EC_*, structures of AmpC*_EC_* in complex with AVI (1.51 Å resolution; PDB entry 6TBW), REL (1.72 Å resolution; PDB entry 6TPM), NAC (1.47 Å resolution; PDB entry 6T7L), and ZID (1.30 Å resolution; PDB entry 6T5Y) were solved. The complexes were obtained using relatively short (10- to 15-min) inhibitor soakings of AmpC*_EC_* crystals, which were produced as reported previously (34). In all cases, there was one protein molecule in each asymmetric unit; structures were solved by molecular replacement, using the reported AmpC*_EC_* structure (PDB entry 6T3D [34]).

**FIG 3:**
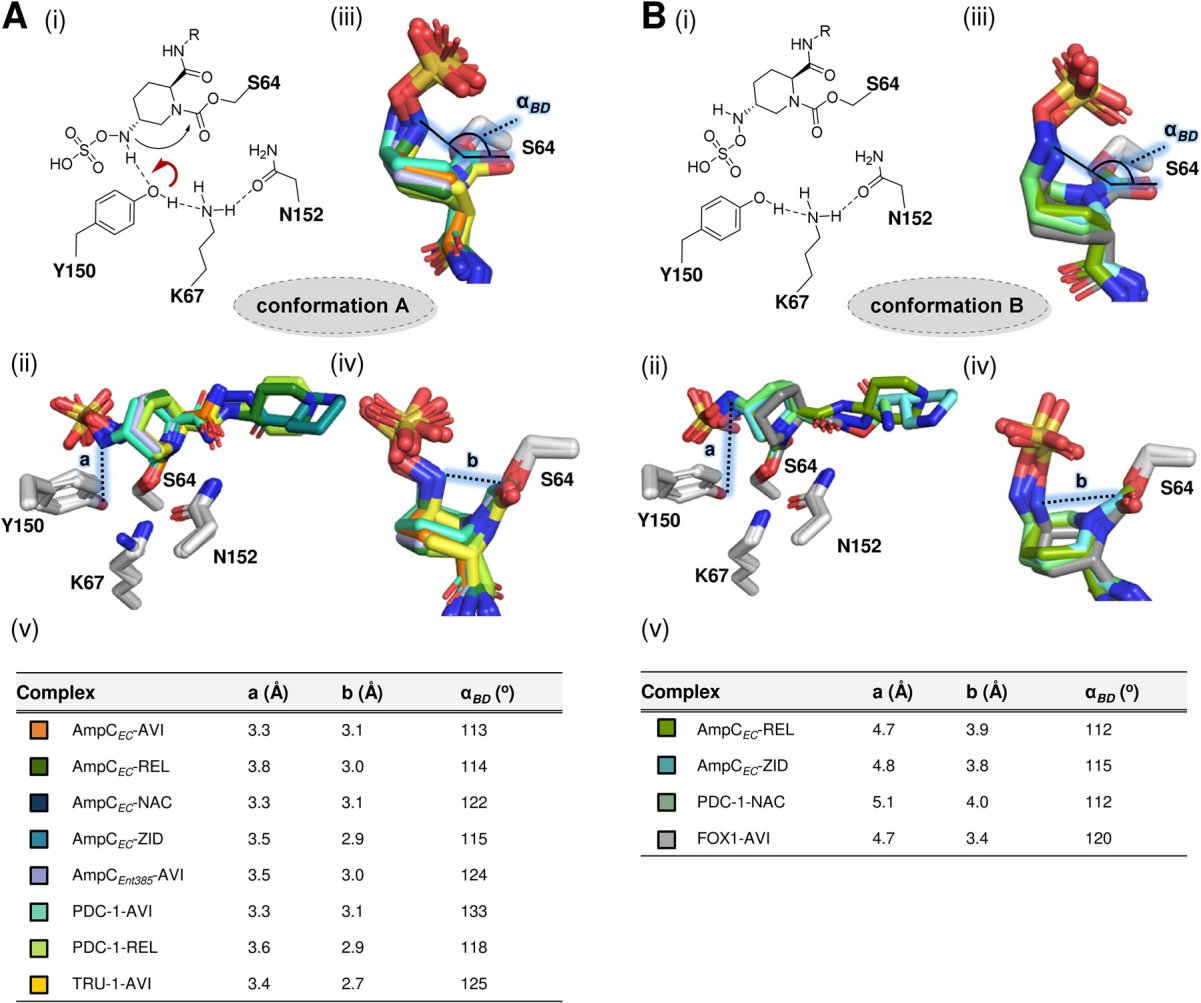
Comparison of DBO-derived AmpC*_EC_* complexes with DBO complexes of other class C SBLs. (A) Conformation A. (B) Conformation B. In each case, the panels show the following details. (i) Schemes showing that in conformation A, the *N*-sulfate nitrogen is proximal to Y150:O_η_ and is favorably arranged for DBO formation, while in conformation B, the *N*-sulfate nitrogen is oriented away from Y150:O_η_ and is not favorably arranged for DBO formation. (ii) Distances (a) between the DBO *N*-sulfate nitrogen and Y150:O_η_. (iii) Bürgi-Dunitz angle (α*_BD_*) for the *N*-sulfate nitrogen and the C=O of the carbonyl group. (iv) Distances (b) between carbonyl-carbon and *N*-sulfate nitrogen. (v) Distances and angles. PDB entries were the following: AmpC_Ent385_-AVI (55) (6LC8), PDC-1-AVI (29) (4OOY), PDC-1-REL (20) (4NK3), TRU-1-AVI (28) (6FM7), PDC-1-NAC (18) (4X68), and FOX-1-AVI (27) (5ZA2). In the structure of the AmpC_Ent385_-AVI complex, K67 is observed in two conformations; in one it is not positioned to interact with N152 (55).

**FIG 4:**
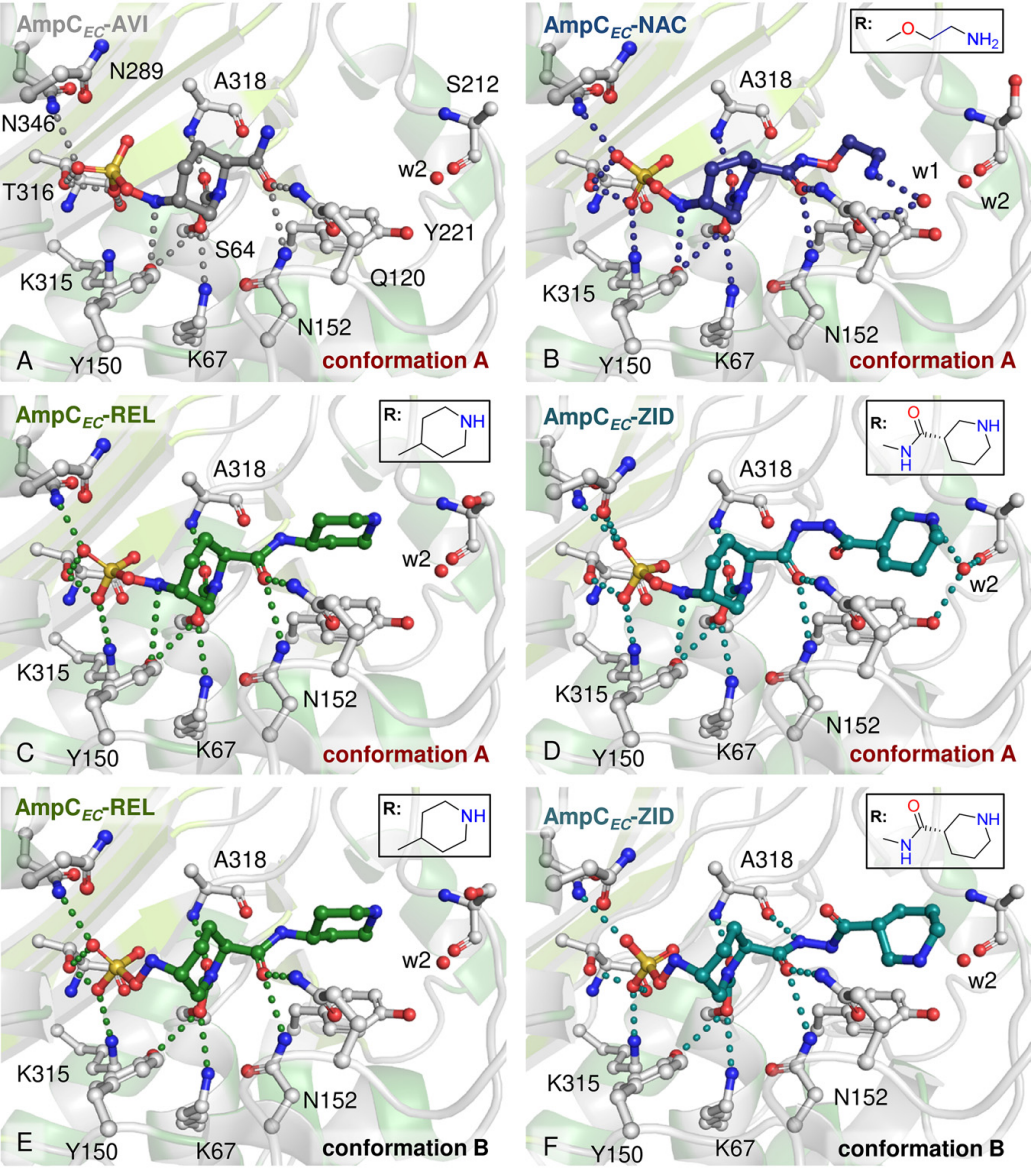
Views of active sites of the complexes of AmpC*_EC_* with AVI (A), NAC (B), REL (C and E), and ZID (D and F). AVI and NAC were modeled in the single conformation A, where the *N*-sulfate nitrogen is proximal to Y150. REL and ZID were modeled in both *N*-sulfate conformations A and B. With ZID, two conformations of the acyl amino sidechain were modeled. Hydrogen bonds are colored balls. Structures of DBOs in their open form, bound to the active-site serine, are in Fig. 2; acyl amido sidechains are in boxes. Polder omit maps (56) and overlays of AVI and ZID conformations A and B are in Fig. S8. Stereo views of all panels are in Fig. S9 to S11.

No changes in the overall fold of AmpC*_EC_* were observed on DBO binding (backbone root mean square deviations of 0.12 Å [AmpC*_EC_*-AVI], 0.14 Å [AmpC*_EC_*-REL], 0.11 Å [AmpC*_EC_*-NAC], and 0.15 Å [AmpC*_EC_*-ZID] compared to apo-AmpC*_EC_* [PDB entry 6T3D (34)]). In all cases, analysis of electron density maps revealed modification of the nucleophilic S64 with no substantial changes in the conformations of active-site residues being observed. The DBOs all reacted to form ring-opened products, with the piperidine ring adopting a chair conformation (Fig. 3 and 4). The carbamoyl-carbonyl oxygen is positioned to interact with the backbone NH of A318 (2.8 to 2.9 Å). The *N*-sulfate group is anchored in the active site via polar interactions with N346 (2.6 to 3.0 Å), T316 (2.3 to 2.7 Å), and K315 (2.8 to 3.1 Å; except for AVI); a detailed summary of active-site interactions is given in Table S7. It should be noted that AmpC-mediated resistance to AVI has been reported to arise from mutations leading to substitutions in this conserved sulfate-binding pocket (29, 42). These mutants are also likely to confer resistance to REL, NAC, and ZID. However, resistance to AVI can also arise from mutations to the Ω-loop and the H-10 helix (29, 43–45). The Ω-loop and H-10 helix are located at the R2 binding pocket of the active site, and the different acyl-amido sidechains of REL, NAC, and ZID, which are positioned in this pocket, are likely to have an impact on inhibition of those AVI-resistant mutants.

In reported structures of AmpC-DBO complexes, the orientation of the *N*-sulfate nitrogen can adopt two conformations. In one (conformation A; Fig. 3 and 4), the *N*-sulfate nitrogen is close to the Y150 phenol sidechain, while in the other (conformation B; Fig. 3 and 4) the nitrogen atom points toward the opposite face of the active site. For class C SBLs, acylation and recyclization are proposed to be catalyzed by Y150, K67, and N152 (Fig. 1D) (29). In conformation A, the *N*-sulfate nitrogen is positioned for stereoelectronically favored reaction (in part, as judged by the Bürgi-Dunitz angle [*α_BD_*] [46] of the *N*-sulfate nitrogen position and the distance to the carbamoyl carbon, Fig. 3 iii and iv). In contrast, in conformation B, the *N*-sulfate nitrogen lone pair is predicted to be positioned away from the carbamoyl carbonyl group. AVI has been observed in conformation A in complex with chromosomally encoded Pseudomonas aeruginosa-derived cephalosporinase (PDC-1; PDB entry 4OOY [29]) as well as with the Enterobacter cloacae AmpC*_Ent385_* and the plasmid-encoded TRU-1 (PDB entry 6FM7 [28]) (Fig. 3A). AVI is observed in conformation B with the plasmid-encoded FOX-4 (PDB entry 5ZA2 [27]) (Fig. 3B). With PDC-1, REL is observed in conformation A (PDB entry 4NK3 [22]; Fig. 3A) and NAC in conformation B (PDB entry 4X68 [18]) (Fig. 3B).

In the AmpC*_EC_*-AVI complex structure, ring-opened AVI is observed in conformation A, with the *N*-sulfate nitrogen positioned proximal to the phenolic oxygen of Y150 (Y150:O_η_, 3.3 Å) and the carbamoyl-carbon (3.1 Å, *α_BD_* = 113°). Interestingly, of the other three DBOs, only reacted NAC is observed solely in conformation A, where its *N*-sulfate nitrogen is 3.3 Å from Y150:O_η_ and 3.1 Å from the carbamoyl carbon; however, the *α_BD_* is 122°, which deviates from the preferred *α_BD_* of ∼107° (46). For REL and ZID, both conformations A and B were observed and refined in equal occupancy. The *N*-sulfate nitrogen is 3.8 and 3.5 Å from Y150:O_η_ in conformation A and 4.7 and 4.8 Å from Y150:O_η_ in conformation B for REL and ZID, respectively (Fig. 3). While the *α_BD_* angles are comparable in both conformations A and B (112° to 115°), the orientation of the *N*-sulfate nitrogen is stereoelectronically unfavorable for reaction with the carbamoyl carbonyl group in conformation B, where the distance to the carbamoyl carbon is significantly increased compared to that of conformation A (3.9 Å compared to 3.0 Å for REL and 3.8 Å compared to 2.8 Å for ZID; Fig. 3 and Table S7). It should be noted that in the refined structures in all four cases, there is evidence for relatively weak electron density close to the DBO sulfate; this indicates low occupancy (likely <10%) of an alternative sulfate position; however, due to its low intensity, assignment was not possible.

Based on the proposed decarbamoylation mechanisms of DBOs for class C SBLs (Fig. 1D) (29) and stereoelectronic considerations of the mechanism of the nucleophilic attack, the REL- and ZID-derived complexes appear less primed for recyclization, since they form both conformations A and B, than those of AVI and NAC, where only conformation A is observed. Thus, the structural observations do not obviously correlate with the accelerated dissociation of ZID compared to AVI, REL, and NAC in solution (Table 1). However, it should be noted that the crystallographically observed binding modes may not accurately represent those in solution, where conformations A and B may be in dynamic equilibrium.

The amide or acyl amido sidechains of all the DBOs interact with the sidechains of N152 (3.0 to 3.2 Å) and Q120 (2.8 to 3.1 Å) in a manner similar to that for the C7 acyl amido sidechains of cephalosporins, e.g., as observed in an AmpC*_EC_*-cephalothin acyl-enzyme complex (PDB entry 1KVL) (47). There are variations in the nature of other aspects of sidechain interactions, although the relevance of these to potency is unclear.

The REL piperidyl sidechain adopts a single conformation and is oriented in a manner similar to that observed in the complex with the *Pseudomonas-*derived cephalosporinase 1 (PDC-1) (20) (PDB entry 4NK3), albeit in a slightly rotated manner (∼30°; Fig. S10). The piperidyl nitrogen does not appear to engage in any direct or water-mediated hydrogen bonding interactions with AmpC*_EC_* active-site residues.

Whereas the *N*-sulfate nitrogen of NAC adopts conformation A in the AmpC*_EC_* complex structure, it is observed in conformation B in complex with the class C SBL PDC-1 (PDB entry 4X58; Fig. S10) (18). In the AmpC*_EC_*-NAC complex, the terminal amine of the DBO sidechain has an orientation different from that observed with PDC-1. In the AmpC*_EC_*-NAC complex, the amino sidechain interacts with a water molecule (w1, 2.8 Å; Fig. 4B), which is not observed in the apo-AmpC*_EC_* structure and which is positioned to further interact with the sidechain of N152 (3.1 Å).

In addition to the two conformations (A and B) observed for the *N*-sulfate nitrogen, two conformations were observed for the ZID acyl-hydrazide sidechain (Fig. 4D and F). Thus, there are (at least) four possible conformations. It was not possible to correlate the two sets of conformations; therefore, only two overall conformations were refined. Thus, the binding mode of ZID appears more variable than those for the other DBOs, a factor that might contribute to the relatively increased carbamoylation and decarbamoylation rates for ZID. However, the validity of such a correlation is uncertain, as to date no other structures of ZID in complex with class C SBLs are available. In one sidechain conformation (refined with *N*-sulfate conformation B), the ZID piperidinyl nitrogen of the acyl-hydrazide is positioned to interact with a water molecule (w1, 3.2 Å; Fig. 4D), which in turn interacts with the main-chain carbonyl of S212 (2.6 Å) and the sidechain of Y221 (2.8 Å). The position of w1 is conserved in all AmpC*_EC_*-DBO complex structures reported here as well as in the unreacted AmpC*_EC_* structure. In the second sidechain conformation (refined with *N*-sulfate conformation A), the acyl-hydrazide sidechain more closely resembles the ZID binding mode, as observed at the KPC-2 active site (17) (PDB entry 6B1J; Fig. S8), and does not interact with w1.

### Conclusions.

Following the clinical introduction of AVI as a broad-spectrum class A and C SBL inhibitor in combination with ceftazidime, interest in DBOs both as SBL inhibitors and as antibacterials has grown significantly. Efforts are ongoing in multiple groups to optimize DBOs. Our results provide detailed insight into the mechanism of inhibition of a clinically relevant SBL, AmpC*_EC_*, by four DBOs. They reveal differences in the interactions of differently substituted DBOs with AmpC*_EC_* that may be of general relevance in optimizing the activity of DBOs and other reversibly reacting SBL inhibitors.

All the tested DBOs potently inhibit isolated AmpC*_EC_*. Interestingly, ZID manifests ∼10-fold faster carbamoylation and decarbamoylation rates than the other tested DBOs (Table 1). As supported both by application of established competition assays and by protein-observed MS studies, this results in the increased potency of ZID after short preincubation times (≤1 min), which becomes less prominent on prolonged preincubation times (as monitored over 6 to 24 h) (Fig. S2 and S3). Indeed, the DBOs have nearly equivalent potencies after prolonged preincubation, i.e., under equilibration conditions. Coupled with the results of our MS-based competition experiments (Fig. 2 and Fig. S4 and S5), these observations imply that the equilibrium positions between the unreacted DBOs and the covalent AmpC*_EC_*-DBO complexes are similar for the tested DBOs. The similar potencies observed after equilibration of different DBOs with AmpC*_EC_* suggest that the AmpC*_EC_*-DBO complexes have stabilities similar to those of ring-closed DBOs/uncomplexed AmpC*_EC_* in solution (40, 41, 48). Thus, at least in the case of AmpC*_EC_*, the difference between ZID and the other DBOs lies in the rate of its reaction with and release from AmpC*_EC_*.

Crystallographic analyses for the four DBO-derived complexes reveal that in the cases of AVI and NAC, the DBO-derived complexes (at least predominantly) adopt conformations that appear primed for AVI recyclization (conformation A; Fig. 3 and 4) via nucleophilic attack of the *N*-sulfate nitrogen with the carbonyl group of the carbamoyl complex. However, in the case of the REL- and ZID-derived complexes, only approximately half of the molecules are in the productive conformation (A), while the other half adopt conformation B (Fig. 3 and 4), in which the *N*-sulfate nitrogen lone pair is directed away from the reactive carbonyl group. Thus, the crystallographic studies do not appear to correlate with the different kinetic properties of ZID compared to the other DBOs in solution, and care should be taken in assuming the crystallographically observed conformations reflect those relevant to solution binding. In solution, it is possible that conformations A and B are in dynamic equilibrium. It should be noted that the details of general acid/base catalysis in the reaction of DBOs with AmpC*_EC_* have not been studied in detail. Detailed modeling studies employing quantum mechanics/molecular mechanics-type methods are of interest to study the relevance of the two conformations, in particular conformation B, in solution. If, indeed, conformation B is not productive with respect to DBO recyclization, efforts to optimize its formation relative to conformation A may be productive.

Although the basis of the different kinetics of ZID is unclear, it is notable that while the relatively large ZID sidechain engages in some additional hydrogen-bonding interactions, the available evidence suggests that the overall binding mode of ZID is more flexible than that for the other DBOs with four possible binding modes (conformations A/B and the two sidechain conformations), which are observed in one (conformation A, AVI and NAC) or two (A and B, REL) *N*-sulfate conformations. The relatively high carbamoylation and decarbamoylation rates (and, hence, differences in activation energy) for ZID compared to the other DBOs may relate to the apparently increased flexibility in the AmpC*_EC_*-ZID complex, although further work is required to validate this proposal.

ZID further demonstrated excellent activity that is superior to that of AVI when tested against an engineered AmpC*_EC_*-expressing E. coli strain, likely due in part to its intrinsic antibacterial activity (Table S5). However, the apparent difference in the half-life of the carbamoyl complexes for ZID compared to the other DBOs also may be related to their *in vivo* properties. It is possible that the relatively short residence time of the AmpC*_EC_*-ZID complex hampers activity *in vivo*, where drug and substrate concentrations can fluctuate and resistance mechanisms (e.g., efflux pumps) may increase the importance of the residency times of the target-inhibitor complex (40, 41, 48). Thus, we propose that there is scope for optimization of the active-site residency times of DBOs in general and suggest that assays to assess this are important in medicinal chemistry efforts on DBOs.

## MATERIALS AND METHODS

### Materials.

DBO derivatives were from commercial suppliers (AVI [AstraZeneca], REL [MedKoo Biosciences, Inc.], NAC [Advanced ChemBlocks, Inc.], and ZID [MedKoo Biosciences, Inc.]). Recombinant AmpC*_EC_* was produced as previously described (34).

### Kinetic studies.

Assays were performed and analyzed as described previously (34). In summary, assays in the presence of inhibitors were carried out under steady-state conditions monitoring hydrolysis of the fluorescent substrate FC-5 (33) in 100 mM phosphate buffer, pH 7.5, supplemented with 0.01% (vol/vol) Triton X-100. Fluorescence was measured using a PHERAstar (BMG Labtech) plate reader, recording emission spectra at an excitation wavelength of 380 nm and emission wavelength of 460 nm. Data were analyzed using Prism 5 (GraphPad Software). pIC_50_s were determined after various incubation times (0, 10, 30, 60, and 360 min) via nonlinear regression (see Fig. S2 in the supplemental material). The pseudo first-order rate constant, *k*_obs_, and the apparent inhibition constant, *K*_iapp_, were obtained by assaying 100 nM AmpC*_EC_* with 5 μM FC-5 in the presence of various concentrations of the inhibitor. Reactions were initiated by addition of AmpC*_EC_* and immediately monitored continuously over 30 s. Activity was fitted to an exponential course in relation to the uninhibited enzyme and a no-enzyme control to give initial rate constants (*k*_obs_) (Fig. S1 and Table S2), as described previously (49). The second-order rate constant (*k*_2_/*K*) was obtained from linear regression of measured *k*_obs_ values against inhibitor concentrations (see Fig. S3b in the supplemental material) and corrected by the Michaelis constant (*K_m_*) and concentration of the substrate. *K*_iapp_s (50) were determined by linear regression of the reciprocals of the initial rates against inhibitor concentration (Fig. S3a) and corrected using the experimentally determined substrate *K_m_* and substrate concentration. Inhibitor off rates (*k*_off_) were measured using the jump-dilution method (51); thus, AmpC*_EC_* (1 μM) was incubated with the respective inhibitor (10 μM) for 30 min at room temperature and diluted 100,000-fold in the assay buffer to a final enzyme concentration of 10 pM and then immediately assayed using 25 μM (final concentration) FC-5. Off rates were determined from exponential regression in relation to turnover of no enzyme and no inhibitor controls (Fig. S3c). Half-lives of the enzyme-inhibitor complexes were directly calculated from the measured *k*_off_ values.

### MICs.

MICs were determined by broth microdilutions in triplicate and interpreted using published guidelines described by the CLSI. CAZ was tested alone (0.25 to 256 μg ml^−1^) against DH5α Escherichia coli and DH5α E. coli containing the pAD7-AmpC*_EC_* plasmid and in combination with AVI (0.125, 0.25, 0.5, 1, 2, 4, or 8 μg ml^−1^), REL (4 μg ml^−1^), NAC (4 μg ml^−1^), and ZID (0.125, 0.25, 0.5, 1, 2, 4, or 8 μg ml^−1^) using the DH5α E. coli strain containing the pAD7-AmpC*_EC_* plasmid (35) (Table S5). DBOs were also tested as the sole administered antimicrobial (0.25 to 256 μg ml^−1^) against both strains (Table S5).

### Protein SPE-electrospray ionization-MS spectrometry.

AmpC*_EC_* (3 μM) in 50 mM Tris, pH 7.5, was incubated with avibactam, relebactam, nacubactam, or zidebactam (all at 3.3 μM) at room temperature. Mass spectra were acquired in the positive ion mode using an integrated autosampler/solid-phase extraction (SPE) RapidFire365 system (Agilent Technologies) coupled to an Agilent 6550 accurate mass QTOF mass spectrometer. After the indicated time (Fig. 3 and Fig. S4), 50 μl of the solution was loaded onto a C4 SPE cartridge (Agilent Technologies); the cartridge was then washed with buffer A (0.1% [vol/vol] aqueous formic acid) and then eluted into the mass spectrometer in buffer B (15% [vol/vol] water, 85% [vol/vol] acetonitrile, 0.1% [vol/vol] formic acid). The cartridge was reequilibrated with buffer A between samples. Data were analyzed using MassHunter Qualitative Analysis software V.7 (Agilent Technologies) using the maximum entropy deconvolution algorithm.

### Protein crystallization and inhibitor soaking.

AmpC*_EC_* was crystallized (space group *P*4_3_32, 1 molecule per asymmetric unit) in its apo form as described previously (34). For soaking experiments, crystals were transferred into a well solution supplemented with AVI, REL, NAC, or ZID and then incubated at room temperature for 10, 6, 8, or 10 min, respectively, cryo-cooled, and stored in liquid nitrogen. Data sets from single crystals were collected using the i03 MX beamline at the Diamond Light Source (Table S1). Structures were solved by molecular replacement in Phaser (52) using the structure of apo-AmpC*_EC_* (PDB entry 6T3D [34]) as the starting model. Alternating cycles of refinement using PHENIX (53) and model building using Coot (54) were performed until *R*_work_ and *R*_free_ converged.

### Data availability.

Coordinates and structure factors of AmpC*_EC_* crystal structures have been deposited in the Protein Data Bank. PDB entries are 6TBW, 6TPM, 6T7L, and 6T5Y for crystal structures of AmpC from Escherichia coli in complex with AVI, REL, NAC, and ZID, respectively.

## Supplementary Material

Supplemental file 1

## References

[1] Croxen MA, Finlay BB 2010 Molecular mechanisms of *Escherichia coli* pathogenicity. Nat Rev Microbiol 8:26–38. doi:10.1038/nrmicro2265. 19966814

[2] Croxen MA, Law RJ, Scholz R, Keeney KM, Wlodarska M, Finlay BB 2013 Recent advances in understanding enteric pathogenic *Escherichia coli*. Clin Microbiol Rev 26:822–880. doi:10.1128/CMR.00022-13. 24092857 PMC3811233

[3] Kaper JB, Nataro JP, Mobley HL 2004 Pathogenic *Escherichia coli*. Nat Rev Microbiol 2:123–140. doi:10.1038/nrmicro818. 15040260

[4] Bush K 2018 Past and present perspectives on β-lactamases. Antimicrob Agents Chemother 62:e01076-18. doi:10.1128/AAC.01076-18. 30061284 PMC6153792

[5] Bush K, Jacoby GA 2010 Updated functional classification of β-lactamases. Antimicrob Agents Chemother 54:969–976. doi:10.1128/AAC.01009-09. 19995920 PMC2825993

[6] Reading C, Cole M 1977 Clavulanic acid: a β-lactamase-inhibiting β-lactam from *Streptomyces clavuligerus*. Antimicrob Agents Chemother 11:852–857. doi:10.1128/aac.11.5.852. 879738 PMC352086

[7] English AR, Retsema JA, Girard AE, Lynch JE, Barth WE 1978 CP-45,899, a β-lactamase inhibitor that extends the antibacterial spectrum of β-lactams: initial bacteriological characterization. J Antimicrob Chemother 14:414–419. doi:10.1128/AAC.14.3.414. PMC352474 309306

[8] Aronoff SC, Jacobs MR, Johenning S, Yamabe S 1984 Comparative activities of the β-lactamase inhibitors YTR 830, sodium clavulanate, and sulbactam combined with amoxicillin or ampicillin. Antimicrob Agents Chemother 26:580–582. doi:10.1128/aac.26.4.580. 6097169 PMC179968

[9] Drawz SM, Bonomo RA 2010 Three decades of β-lactamase inhibitors. Clin Microbiol Rev 23:160–201. doi:10.1128/CMR.00037-09. 20065329 PMC2806661

[10] Jacoby GA 2009 AmpC β-lactamases. Clin Microbiol Rev 22:161–182. doi:10.1128/CMR.00036-08. 19136439 PMC2620637

[11] Jaurin B, Grundström T, Edlund T, Normark S 1981 The *E. coli* β-lactamase attenuator mediates growth rate-dependent regulation. Nature 290:221–225. doi:10.1038/290221a0. 7010184

[12] Nelson EC, Elisha BG 1999 Molecular basis of AmpC hyperproduction in clinical isolates of *Escherichia coli*. Antimicrob Agents Chemother 43:957–959. doi:10.1128/AAC.43.4.957. 10103209 PMC89235

[13] Wang DY, Abboud MI, Markoulides MS, Brem J, Schofield CJ 2016 The road to avibactam: the first clinically useful non-β-lactam working somewhat like a β-lactam. Future Med Chem 8:1063–1084. doi:10.4155/fmc-2016-0078. 27327972

[14] Ehmann DE, Jahic H, Ross PL, Gu RF, Hu J, Kern G, Walkup GK, Fisher SL 2012 Avibactam is a covalent, reversible, non-β-lactam β-lactamase inhibitor. Proc Natl Acad Sci U S A 109:11663–11668. doi:10.1073/pnas.1205073109. 22753474 PMC3406822

[15] Ehmann DE, Jahic H, Ross PL, Gu RF, Hu J, Durand-Reville TF, Lahiri S, Thresher J, Livchak S, Gao N, Palmer T, Walkup GK, Fisher SL 2013 Kinetics of avibactam inhibition against Class A, C, and D β-lactamases. J Biol Chem 288:27960–27971. doi:10.1074/jbc.M113.485979. 23913691 PMC3784710

[16] Choi H, Paton RS, Park H, Schofield CJ 2016 Investigations on recyclisation and hydrolysis in avibactam mediated serine β-lactamase inhibition. Org Biomol Chem 14:4116–4128. doi:10.1039/c6ob00353b. 27072755 PMC4847122

[17] Papp-Wallace KM, Nguyen NQ, Jacobs MR, Bethel CR, Barnes MD, Kumar V, Bajaksouzian S, Rudin SD, Rather PN, Bhavsar S, Ravikumar T, Deshpande PK, Patil V, Yeole R, Bhagwat SS, Patel MV, van den Akker F, Bonomo RA 2018 Strategic approaches to overcome resistance against gram-negative pathogens using β-lactamase inhibitors and β-lactam enhancers: activity of three novel diazabicyclooctanes WCK 5153, zidebactam (WCK 5107), and WCK 4234. J Med Chem 61:4067–4086. doi:10.1021/acs.jmedchem.8b00091. 29627985 PMC6131718

[18] Morinaka A, Tsutsumi Y, Yamada M, Suzuki K, Watanabe T, Abe T, Furuuchi T, Inamura S, Sakamaki Y, Mitsuhashi N, Ida T, Livermore DM 2015 OP0595, a new diazabicyclooctane: mode of action as a serine β-lactamase inhibitor, antibiotic and β-lactam ‘enhancer’. J Antimicrob Chemother 70:2779–2786. doi:10.1093/jac/dkv166. 26089439

[19] Moya B, Barcelo IM, Bhagwat S, Patel M, Bou G, Papp-Wallace KM, Bonomo RA, Oliver A 2017 WCK 5107 (Zidebactam) and WCK 5153 are novel inhibitors of PBP2 showing potent “β-Lactam Enhancer” activity against *Pseudomonas aeruginosa*, including multidrug-resistant metallo-β-lactamase-producing high-risk clones. Antimicrob Agents Chemother 61:e02529-16. doi:10.1128/AAC.02529-16. 28289035 PMC5444176

[20] Blizzard TA, Chen H, Kim S, Wu J, Bodner R, Gude C, Imbriglio J, Young K, Park Y-W, Ogawa A, Raghoobar S, Hairston N, Painter RE, Wisniewski D, Scapin G, Fitzgerald P, Sharma N, Lu J, Ha S, Hermes J, Hammond ML 2014 Discovery of MK-7655, a β-lactamase inhibitor for combination with Primaxin. Bioorg Med Chem Lett 24:780–785. doi:10.1016/j.bmcl.2013.12.101. 24433862

[21] Durand-Reville TF, Guler S, Comita-Prevoir J, Chen B, Bifulco N, Huynh H, Lahiri S, Shapiro AB, McLeod SM, Carter NM, Moussa SH, Velez-Vega C, Olivier NB, McLaughlin R, Gao N, Thresher J, Palmer T, Andrews B, Giacobbe RA, Newman JV, Ehmann DE, de Jonge B, O'Donnell J, Mueller JP, Tommasi RA, Miller AA 2017 ETX2514 is a broad-spectrum β-lactamase inhibitor for the treatment of drug-resistant Gram-negative bacteria including *Acinetobacter baumannii*. Nat Microbiol 2:17104. doi:10.1038/nmicrobiol.2017.104. 28665414

[22] King DT, King AM, Lal SM, Wright GD, Strynadka NCJ 2015 Molecular mechanism of avibactam-mediated β-lactamase inhibition. ACS Infect Dis 1:175–184. doi:10.1021/acsinfecdis.5b00007. 27622530

[23] Krishnan NP, Nguyen NQ, Papp-Wallace KM, Bonomo RA, van den Akker F 2015 Inhibition of Klebsiella β-lactamases (SHV-1 and KPC-2) by avibactam: a structural study. PLoS One 10:e0136813. doi:10.1371/journal.pone.0136813. 26340563 PMC4560403

[24] Lahiri SD, Mangani S, Durand-Reville T, Benvenuti M, De Luca F, Sanyal G, Docquier J-D 2013 Structural insight into potent broad-spectrum inhibition with reversible recyclization mechanism: avibactam in complex with CTX-M-15 and *Pseudomonas aeruginosa* AmpC β-lactamases. Antimicrob Agents Chemother 57:2496–2505. doi:10.1128/AAC.02247-12. 23439634 PMC3716117

[25] Lahiri SD, Mangani S, Jahić H, Benvenuti M, Durand-Reville TF, De Luca F, Ehmann DE, Rossolini GM, Alm RA, Docquier J-D 2015 Molecular basis of selective inhibition and slow reversibility of avibactam against class D carbapenemases: a structure-guided study of OXA-24 and OXA-48. ACS Chem Biol 10:591–600. doi:10.1021/cb500703p. 25406838

[26] Lohans CT, Wang DY, Jorgensen C, Cahill ST, Clifton IJ, McDonough MA, Oswin HP, Spencer J, Domene C, Claridge TDW, Brem J, Schofield CJ 2017 13C-carbamylation as a mechanistic probe for the inhibition of class D β-lactamases by avibactam and halide ions. Org Biomol Chem 15:6024–6032. doi:10.1039/c7ob01514c. 28678295

[27] Nukaga M, Papp-Wallace KM, Hoshino T, Lefurgy ST, Bethel CR, Barnes MD, Zeiser ET, Johnson JK, Bonomo RA 2018 Probing the Mechanism of Inactivation of the FOX-4 Cephamycinase by Avibactam. Antimicrob Agents Chemother 62:e02371-17. doi:10.1128/AAC.02371-17. 29439972 PMC5923096

[28] Pozzi C, Di Pisa F, De Luca F, Benvenuti M, Docquier JD, Mangani S 2018 Atomic-resolution structure of a class C β-lactamase and its complex with avibactam. ChemMedChem 13:1437–1446. doi:10.1002/cmdc.201800213. 29786960

[29] Lahiri SD, Johnstone MR, Ross PL, McLaughlin RE, Olivier NB, Alm RA 2014 Avibactam and class C β-lactamases: mechanism of inhibition, conservation of the binding pocket, and implications for resistance. Antimicrob Agents Chemother 58:5704–5713. doi:10.1128/AAC.03057-14. 25022578 PMC4187909

[30] Jin W, Wachino J-I, Yamaguchi Y, Kimura K, Kumar A, Yamada M, Morinaka A, Sakamaki Y, Yonezawa M, Kurosaki H, Arakawa Y 2017 Structural insights into the TLA-3 extended-spectrum β-lactamase and its inhibition by avibactam and OP0595. Antimicrob Agents Chemother 61:e00501-17. doi:10.1128/AAC.00501-17. 28739781 PMC5610492

[31] Ruggiero M, Papp-Wallace KM, Brunetti F, Barnes MD, Bonomo RA, Gutkind G, Klinke S, Power P 2019 Structural insights into the inhibition of the extended-spectrum β-lactamase PER-2 by avibactam. Antimicrob Agents Chemother 63:e00487-19. doi:10.1128/AAC.00487-19. 31235626 PMC6709463

[32] Tooke CL, Hinchliffe P, Lang PA, Mulholland AJ, Brem J, Schofield CJ, Spencer J 2019 Molecular basis of class A β-lactamase inhibition by relebactam. Antimicrob Agents Chemother 63:e00564-19. doi:10.1128/AAC.00564-19. 31383664 PMC6761529

[33] van Berkel SS, Brem J, Rydzik AM, Salimraj R, Cain R, Verma A, Owens RJ, Fishwick CWG, Spencer J, Schofield CJ 2013 Assay platform for clinically relevant metallo-β-lactamases. J Med Chem 56:6945–6953. doi:10.1021/jm400769b. 23898798 PMC3910272

[34] Lang PA, Parkova A, Leissing TM, Calvopina K, Cain R, Krajnc A, Panduwawala TD, Philippe J, Fishwick CW, Trapencieris P, Page MGP, Schofield CJ, Brem J 2020 Bicyclic boronates as potent inhibitors of AmpC, the class C β-lactamase from *Escherichia coli*. Biomolecules 10:899. doi:10.3390/biom10060899. PMC7356297 32545682

[35] Dubus A, Wilkin JM, Raquet X, Normark S, Frère JM 1994 Catalytic mechanism of active-site serine β-lactamases: role of the conserved hydroxy group of the Lys-Thr(Ser)-Gly triad. Biochem J 301:485–494. doi:10.1042/bj3010485. 8042993 PMC1137107

[36] Reck F, Bermingham A, Blais J, Casarez A, Colvin R, Dean CR, Furegati M, Gamboa L, Growcott E, Li C, Lopez S, Metzger L, Nocito S, Ossola F, Phizackerley K, Rasper D, Shaul J, Shen X, Simmons RL, Tang D, Tashiro K, Yue Q 2019 IID572: a new potentially best-in-class β-lactamase inhibitor. ACS Infect Dis 5:1045–1051. doi:10.1021/acsinfecdis.9b00031. 30861342

[37] Asli A, Brouillette E, Krause KM, Nichols WW, Malouin F 2016 Distinctive binding of avibactam to penicillin-binding proteins of gram-negative and gram-positive bacteria. Antimicrob Agents Chemother 60:752–756. doi:10.1128/AAC.02102-15. 26574008 PMC4750707

[38] Hayes MV, Orr DC 1983 Mode of action of ceftazidime: affinity for the penicillin-binding proteins of *Escherichia coli* K12, *Pseudomonas aeruginosa* and *Staphylococcus aureus*. J Antimicrob Chemother 12:119–126. doi:10.1093/jac/12.2.119. 6413485

[39] Livermore DM, Mushtaq S, Warner M, Woodford N 2015 Activity of OP0595/β-lactam combinations against Gram-negative bacteria with extended-spectrum, AmpC and carbapenem-hydrolysing β-lactamases. J Antimicrob Chemother 70:3032–3041. doi:10.1093/jac/dkv239. 26311835

[40] Tonge PJ 2018 Drug–target kinetics in drug discovery. ACS Chem Neurosci 9:29–39. doi:10.1021/acschemneuro.7b00185. 28640596 PMC5767540

[41] Lu H, Tonge PJ 2010 Drug-target residence time: critical information for lead optimization. Curr Opin Chem Biol 14:467–474. doi:10.1016/j.cbpa.2010.06.176. 20663707 PMC2918722

[42] Compain F, Debray A, Adjadj P, Dorchêne D, Arthur M 2020 Ceftazidime-avibactam resistance mediated by the N_346_Y substitution in various AmpC β-lactamases. Antimicrob Agents Chemother 64:e02311-19. doi:10.1128/AAC.02311-19. 32253219 PMC7269493

[43] Lahiri SD, Walkup GK, Whiteaker JD, Palmer T, McCormack K, Tanudra MA, Nash TJ, Thresher J, Johnstone MR, Hajec L, Livchak S, McLaughlin RE, Alm RA 2015 Selection and molecular characterization of ceftazidime/avibactam-resistant mutants in *Pseudomonas aeruginosa* strains containing derepressed AmpC. J Antimicrob Chemother 70:1650–1658. doi:10.1093/jac/dkv004. 25645206

[44] Livermore DM, Mushtaq S, Doumith M, Jamrozy D, Nichols WW, Woodford N 2018 Selection of mutants with resistance or diminished susceptibility to ceftazidime/avibactam from ESBL- and AmpC-producing Enterobacteriaceae. J Antimicrob Chemother 73:3336–3345. doi:10.1093/jac/dky363. 30247546

[45] Russ D, Glaser F, Shaer Tamar E, Yelin I, Baym M, Kelsic ED, Zampaloni C, Haldimann A, Kishony R 2020 Escape mutations circumvent a tradeoff between resistance to a β-lactam and resistance to a β-lactamase inhibitor. Nat Commun 11:2029. doi:10.1038/s41467-020-15666-2. 32332717 PMC7181632

[46] Bürgi HB, Dunitz JD, Lehn JM, Wipff G 1974 Stereochemistry of reaction paths at carbonyl centres. Tetrahedron 30:1563–1572. doi:10.1016/S0040-4020(01)90678-7.

[47] Beadle BM, Trehan I, Focia PJ, Shoichet BK 2002 Structural milestones in the reaction pathway of an amide hydrolase: substrate, acyl, and product complexes of cephalothin with AmpC β-lactamase. Structure 10:413–424. doi:10.1016/s0969-2126(02)00725-6. 12005439

[48] Copeland RA, Pompliano DL, Meek TD 2006 Drug–target residence time and its implications for lead optimization. Nat Rev Drug Discov 5:730–739. doi:10.1038/nrd2082. 16888652

[49] Morrison JF, Walsh CT 1988 The behavior and significance of slow-binding enzyme inhibitors. Adv Enzymol Relat Areas Mol Biol 61:201–301. doi:10.1002/9780470123072.ch5. 3281418

[50] Williams JW, Morrison JF 1979 The kinetics of reversible tight-binding inhibition. Methods Enzymol 63:437–467. doi:10.1016/0076-6879(79)63019-7. 502865

[51] Copeland RA, Basavapathruni A, Moyer M, Scott MP 2011 Impact of enzyme concentration and residence time on apparent activity recovery in jump dilution analysis. Anal Biochem 416:206–210. doi:10.1016/j.ab.2011.05.029. 21669181

[52] McCoy AJ, Grosse-Kunstleve RW, Adams PD, Winn MD, Storoni LC, Read RJ 2007 Phaser crystallographic software. J Appl Crystallogr 40:658–674. doi:10.1107/S0021889807021206. 19461840 PMC2483472

[53] Adams PD, Grosse-Kunstleve RW, Hung LW, Ioerger TR, McCoy AJ, Moriarty NW, Read RJ, Sacchettini JC, Sauter NK, Terwilliger TC 2002 PHENIX: building new software for automated crystallographic structure determination. Acta Crystallogr D Biol Crystallogr 58:1948–1954. doi:10.1107/s0907444902016657. 12393927

[54] Emsley P, Lohkamp B, Scott WG, Cowtan K 2010 Features and development of Coot. Acta Crystallogr D Biol Crystallogr 66:486–501. doi:10.1107/S0907444910007493. 20383002 PMC2852313

[55] Kawai A, McElheny CL, Iovleva A, Kline EG, Sluis-Cremer N, Shields RK, Doi Y 2020 Structural basis of reduced susceptibility to ceftazidime–avibactam and cefiderocol in *Enterobacter cloacae* due to AmpC R2 loop deletion. Antimicrob Agents Chemother 64:198–200. doi:10.1128/AAC.00198-20. PMC7318025 32284381

[56] Liebschner D, Afonine PV, Moriarty NW, Poon BK, Sobolev OV, Terwilliger TC, Adams PD 2017 Polder maps: improving OMIT maps by excluding bulk solvent. Acta Crystallogr D Struct Biol 73:148–157. doi:10.1107/S2059798316018210. 28177311 PMC5297918

