# Supplementary material for: Structural Investigations of the Inhibition of Escherichia coli AmpC β-Lactamase by Diazabicyclooctanes
Source: Antimicrob Agents Chemother. 2021 Jan 20;65(2):e02073-20. doi: 10.1128/AAC.02073-20 (PMC7849013; doi:10.1128/AAC.02073-20)
Supplement: Supplemental file 1 [file AAC.02073-20-s0001.pdf]

Supplementary Information

Structural Investigations of the Inhibition of *Escherichia coli* AmpC β-Lactamase by Diazabicyclooctanes

Pauline A. Lang[a], Thomas M. Leissing[a], Malcolm G. P. Page[b], Christopher J. Schofield[a,#], Jürgen Brem[a,#]

[a]Department of Chemistry, Chemistry Research Laboratory, University of Oxford, 12 Mansfield Road, Oxford, United Kingdom.

[b]Jacobs University Bremen gGmbH, 28759 Bremen, Germany.

[#]Email: christopher.schofield@chem.ox.ac.uk or jurgen.brem@chem.ox.ac.uk

Table S1: Data collection and Refinement statistics for AmpC$_{EC}$-DBO complex structures.

**Datasets**	**AmpC$_{EC}$-AVI** (PDB ID: 6TBW)	**AmpC$_{EC}$-REL** (PDB ID: 6TPM)	**AmpC$_{EC}$-NAC** (PDB ID: 6T7L)
**Data Collection**			
Beamline (Wavelength, Å)	DLS I03 (0.9763)	DLS I03 (0.9763)	DLS I03 (0.9763)
Detector	Eiger2 XE 16M	Pilatus 6M-F	Pilatus 6M-F
Data Processing	Xia2 dials	Xia2 3dii	Xia2 dials
Space group	$P\,4_3\,3\,2$	$P\,4_3\,3\,2$	$P\,4_3\,3\,2$
Cell dimensions			
a,b,c (Å)	138.1, 138.1, 138.1	138.5, 138.5, 138.5	138.2, 138.2, 138.2
α, β, γ (°)	90, 90, 90	90, 90, 90	90, 90, 90
No. of molecules/ASU	1	1	1
Total reflections	5488176 (253196)*	3750555 (179552)*	5815425 (284851)*
Unique reflections	70726 (3475)*	48671 (2401)*	76716 (4213)*
Resolution (Å)	69.10-1.51 (1.54-1.51)*	56.57-1.72 (1.75-1.72)*	69.09-1.47 (3.99-1.47)*
R_{meas}(I)	0.113 (2.848)*	0.113 (3.785)*	0.134 (3.003)*
I/σI	29.0 (2.6)*	31.6 (1.7)*	25.9 (2.3)*
CC-half	1.0 (0.8)*	1.00 (0.7)*	1.0 (0.8)*
Completeness (%)	100 (100)*	100 (100)*	100 (100)*
Multiplicity	77.6 (72.9)*	77.1 (74.8)*	75.8 (76.3)*
Wilson B value (Å^2)	18.67	29.41	16.23
**Refinement**	PHENIX	PHENIX	PHENIX
R_{work}/R_{free}	0.1584/0.1808	0.1690/0.1953	0.1546/0.1823
No. atoms	3282	3224	3439
- Enzyme	2863	2833	2929
- DBO	17	46	21
- Water	389	313	459
Average B-factors	28.2	38.6	23.4
- Enzyme	26.6	37.4	21.4
- DBO	40.5	57.2	29.5
- Water	39.1	44.3	35.2
R.m.s deviations			
- Bond lengths (Å)	0.008	0.004	0.006
- Bond angles (°)	1.06	0.82	1.27

*Highest resolution shell in parentheses.

Table S1 continued.

**Datasets**	**AmpC$_{EC}$-ZID** (PDB ID: 6T5Y)
**Data Collection**	
Beamline (Wavelength, Å)	DLS I03 (0.9762)
Detector	Pilatus 6M-F
Data Processing	Xia2 dials
Space group	$P\,4_3\,3\,2$
Cell dimensions	
a,b,c (Å)	137.8, 137.8, 137.8
α, β, γ (°)	90, 90, 90
No. of molecules/ASU	1
Total Reflections	8261802 (385630)*
Unique reflections	109248 (5376)*
Resolution (Å)	68.9-1.30 (1.32-1.30)*
R_{meas}(I)	0.085 (0.825)*
I/σI	26.8 (2.4)*
CC-half	1.0 (0.8)*
Completeness (%)	100 (100)*
Multiplicity	75.6 (71.7)*
Wilson B value (Å^2)	13.75
**Refinement**	PHENIX
R_{work}/R_{free}	0.1318/0.1487
No. atoms	3387
- Enzyme	2900
- DBO	52
- Water	419
Average B-factors	21.3
- Enzyme	19.4
- DBO	23.1
- Water	33.6
R.m.s deviations	
- Bond lengths (Å)	0.008
- Bond angles (°)	1.16

*Highest resolution shell in parentheses.

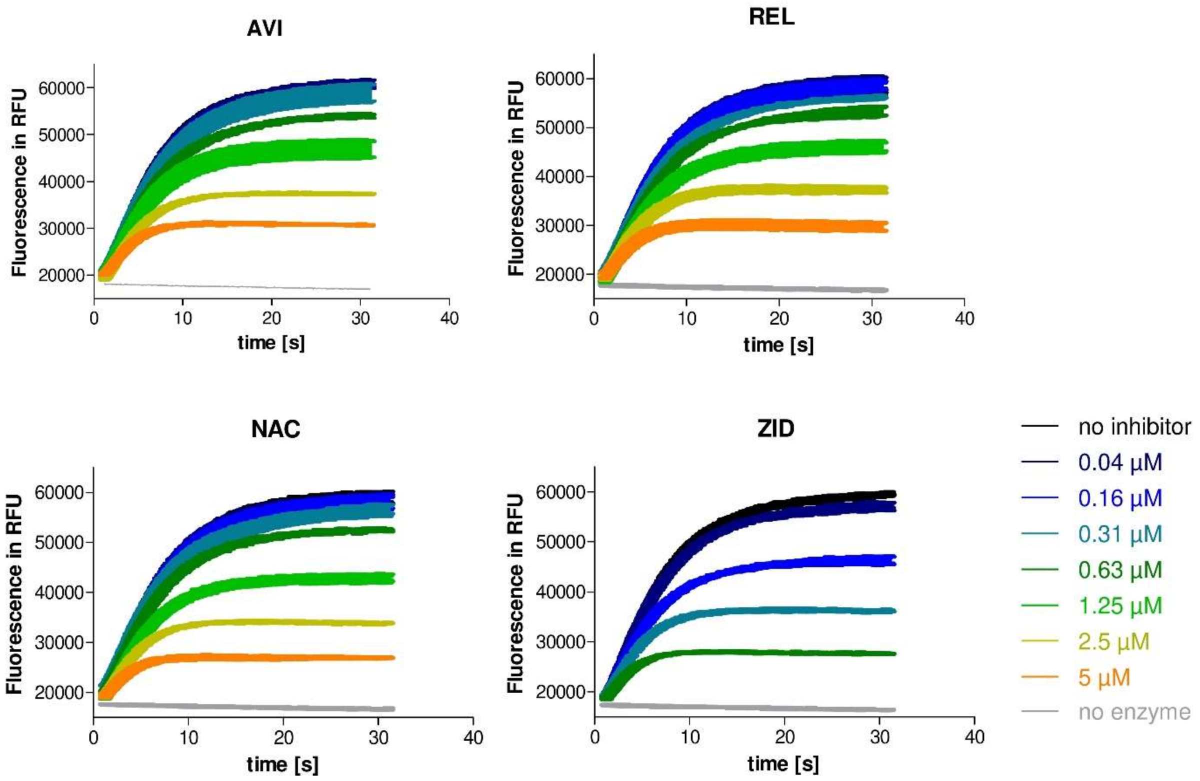

Figure S1: Progression curves used in k_{obs} determinations. AmpC$_{EC}$ (100 nM) was assayed using FC-5 (5 µM) in the presence of AVI, REL, NAC, or ZID at the indicated concentrations. Reactions were initiated by addition of AmpC$_{EC}$ and immediately monitored, as described in Materials and Methods. Buffer: 50 mM phosphate buffer, pH 7.5 and 0.01% (v/v) Triton X-100. Curves were fitted to Equation 5. The resulting k_{obs} values are given in Table S2.

Table S2: k_{obs} values for AmpC$_{EC}$ inhibition by AVI, REL, NAC, and ZID. Values were obtained by fitting the progression curves shown in Figure S1 to Equation 5, as described in Materials and Methods. ND: Not determined.

	k_{obs}			
**[DBO] / µM**	**AVI**	**REL**	**NAC**	**ZID**
**5.00**	0.305 ± 0.004	0.314 ± 0.012	0.393 ± 0.008	*ND*
**2.50**	0.221 ± 0.002	0.226 ± 0.005	0.263 ± 0.002	*ND*
**1.25**	0.168 ± 0.008	0.167 ± 0.005	0.185 ± 0.003	*ND*
**0.63**	0.144 ± 0.002	0.143 ± 0.002	0.153 ± 0.002	0.357 ± 0.003
**0.31**	0.135 ± 0.006	0.136 ± 0.002	0.153 ± 0.005	0.246 ± 0.002
**0.16**	0.134 ± 0.001	0.138 ± 0.003	0.142 ± 0.003	0.183 ± 0.002
**0.04**	0.135 ± 0.002	0.134 ± 0.007	0.142 ± 0.003	0.150 ± 0.002

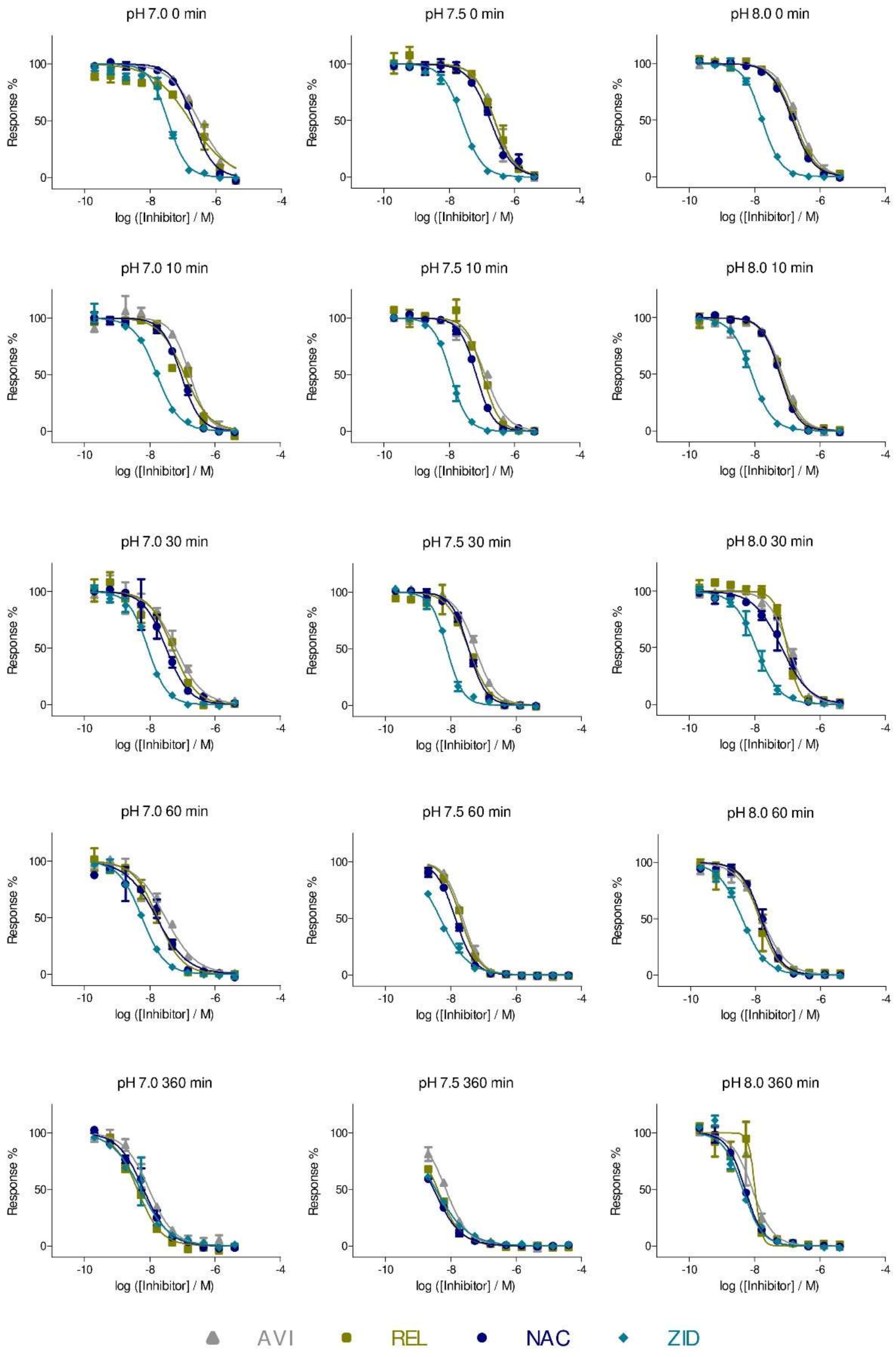

Figure S2: Dose dependence curves for AmpC$_{EC}$ inhibition by AVI, REL, NAC, or ZID. AmpC$_{EC}$ (500 pM) was pre-incubated with the inhibitors for the indicated times at room temperature and assayed using 5 µM FC-5[1]. Buffer: 50 mM phosphate buffer, pH 7.0, 7.5, or 8.0, containing 0.01% (v/v) Triton X-100. pIC$_{50}$s were determined using non-linear regression and are given in Table S3.

Table S3: pH Dependence of $AmpC_{EC}$ inhibition by AVI, REL, NAC or ZID. $AmpC_{EC}$ (500 pM) was preincubated with the inhibitors for the indicated times at room temperature, then assayed using 5 µM FC-5[1]. Buffer: 50 mM phosphate buffer, pH 7.0, 7.5, or 8.0, containing 0.01% (v/v) Triton X-100. pIC_{50}s were determined using non-linear regression (Figure S2), as described in the Materials and Methods. ND: Not determined.

			pIC_{50}	
	**time**	**pH 7.0**	**pH 7.5**	**pH 8.0**
**AVI**	0 min	6.53 ± 0.06	6.62 ± 0.04	6.69 ± 0.02
	10 min	6.78 ± 0.04	6.90 ± 0.03	7.15 ± 0.03
	30 min	7.21 ± 0.08	7.26 ± 0.03	*ND*
	60 min	7.52 ± 0.05	7.64 ± 0.01	7.81 ± 0.04
	360 min	8.02 ± 0.04	8.16 ± 0.02	8.07 ± 0.06
**REL**	0 min	6.78 ± 0.08	6.61 ± 0.04	6.80 ± 0.02
	10 min	6.94 ± 0.05	6.97 ± 0.04	7.20 ± 0.02
	30 min	7.31 ± 0.04	7.46 ± 0.03	*ND*
	60 min	7.76 ± 0.05	7.69 ± 0.02	7.88 ± 0.05
	360 min	8.38 ± 0.03	8.41 ± 0.01	7.98 ± 0.08
**NAC**	0 min	6.70 ± 0.03	6.75 ± 0.04	6.85 ± 0.01
	10 min	7.04 ± 0.03	7.18 ± 0.01	7.23 ± 0.01
	30 min	7.50 ± 0.05	7.46 ± 0.02	*ND*
	60 min	7.79 ± 0.06	7.86 ± 0.02	7.82 ± 0.03
	360 min	8.19 ± 0.04	8.53 ± 0.02	8.29 ± 0.03
**ZID**	0 min	7.46 ± 0.03	7.60 ± 0.02	7.77 ± 0.02
	10 min	7.78 ± 0.02	7.96 ± 0.02	8.09 ± 0.02
	30 min	8.06 ± 0.02	8.12 ± 0.02	*ND*
	60 min	8.24 ± 0.02	8.33 ± 0.02	8.41 ± 0.02
	360 min	8.28 ± 0.07	8.46 ± 0.02	8.36 ± 0.03

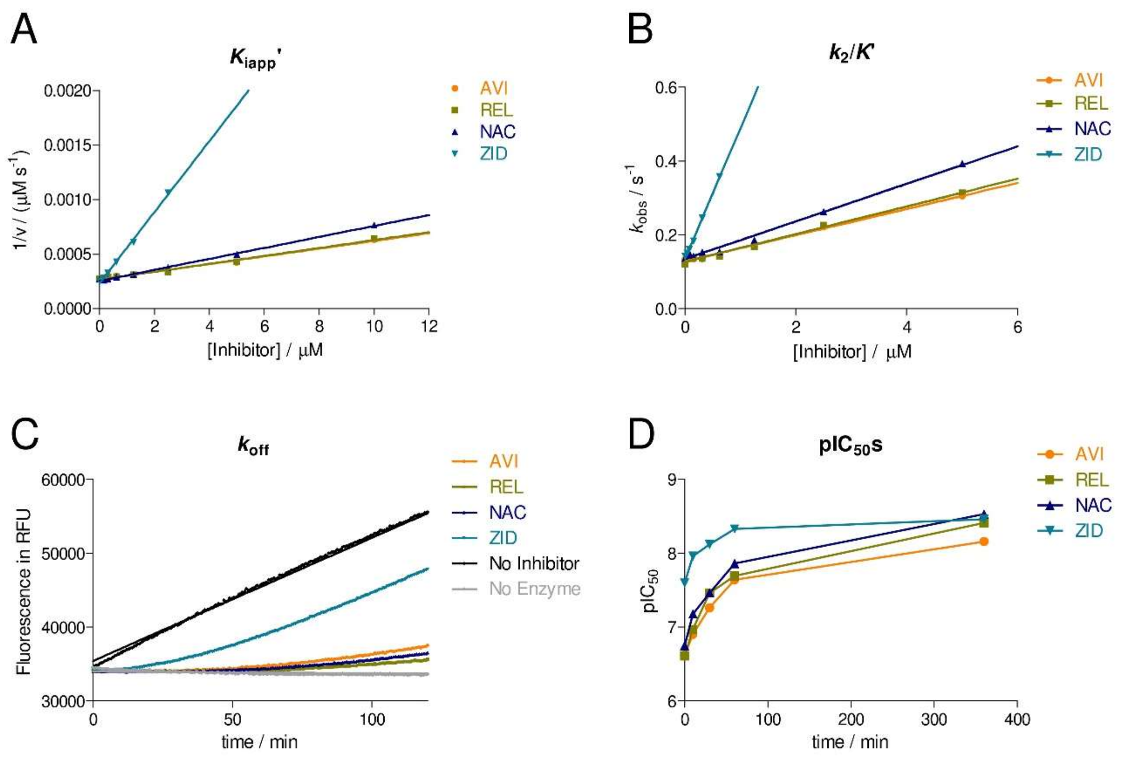

Figure S3: Kinetic characterization of reversible AmpC$_{EC}$ inhibition by DBOs. (A) Determination of K_{iapp} values for AmpC$_{EC}$ inhibition by DBOs. (B) Pseudo first-order rate (k_2/K) determination for AmpC$_{EC}$ (100 nM) with AVI, REL, NAC, or ZID. (C) Dissociation after jump-dilution of AmpC$_{EC}$ (1 µM) pre-incubated with DBOs (10 µM) at room temperature for 30 min, then diluted 100,000 fold and assayed with FC-5[1] (25 µM). (D) Time-dependency of pIC_{50}s determined for AmpC$_{EC}$ (500 pM), assayed using FC-5[1] (5 µM). Buffer: 50 mM Tris, pH 7.5, containing 0.01 % (v/v) Triton-X 100.

Table S4: Summary of kinetic analyses of AmpC$_{EC}$ inhibition by DBOs *in vitro* compared with values reported for PDC-1, PDC-3, and ADC-7[2]. ND: Not determined.

	K_{iapp} (μM)	k_2/K ($M^{-1}s^{-1}$) x 10^3	k_{off} (s^{-1}) x 10^{-3}	$t_{1/2}$ (min)
**AmpC$_{EC}$**				
AVI	7.4 ± 0.3	36 ± 1	0.060 ± 0.01	192 ± 1
REL	7.1 ± 0.3	38 ± 1	0.032 ± 0.01	364 ± 2
NAC	5.0 ± 0.1	52 ± 2	0.049 ± 0.01	236 ± 1
ZID	0.69 ± 0.04	360 ± 10	0.350 ± 0.01	39 ± 4
PDC-1 [2]				
AVI	*ND*	2900	1.9	6
PDC-3 [2]				
AVI	2.5 ± 0.3	29	0.80	14
REL	3.2 ± 0.3	46 ± 5	0.90 ± 0.01	13 ± 4
ZID	0.14 ± 0.01	480 ± 70	3.5 ± 0.4	3 ± 0.4
ADC-7 [2]				
AVI	19 ± 2	3.9 ± 0.4	0.35 ± 0.05	33 ± 5
REL	12.6 ± 2	7.8 ± 0.8	0.30 ± 0.03	39 ± 4
ZID	2.3 ± 0.3	35 ± 4	1.1 ± 0.1	11 ± 1

Table S5: Minimum inhibitory concentrations of DBOs, Ceftazidime, and Ceftazidime – DBO combinations against *E. coli* strains. See Materials and Methods for assay details. ND: Not determined.

	**Minimum Inhibitory Concentration [ug mL^{-1}]**	
**Compound**	**DH5α pAD7 AmpC$_{EC}$**	**DH5α wt**
AVI	16	16
REL	128	128
NAC	4	2
ZID	≤ 0.25	≤ 0.25
CAZ	256	1
CAZ + AVI (0.125 µg mL^{-1})	16	*ND*
CAZ + AVI (0.25 µg mL^{-1})	2	*ND*
CAZ + AVI (0.5 µg mL^{-1})	2	*ND*
CAZ + AVI (1 µg mL^{-1})	0.5	*ND*
CAZ + AVI (2 µg mL^{-1})	0.5	*ND*
CAZ + AVI (4 µg mL^{-1})	0.25	*ND*
CAZ + AVI (8 µg mL^{-1})	0.5	*ND*
CAZ + REL (4 µg mL^{-1})	1	*ND*
CAZ + NAC (4 µg mL^{-1})	≤ 0.25	*ND*
CAZ + ZID (0.125 µg mL^{-1})	2	*ND*
CAZ + ZID (0.25 µg mL^{-1})	1	*ND*
CAZ + ZID (0.5 µg mL^{-1})	≤ 0.25	*ND*
CAZ + ZID (1 µg mL^{-1})	≤ 0.25	*ND*
CAZ + ZID (2 µg mL^{-1})	≤ 0.25	*ND*
CAZ + ZID (4 µg mL^{-1})	≤ 0.25	*ND*
CAZ + ZID (8 µg mL^{-1})	≤ 0.25	*ND*

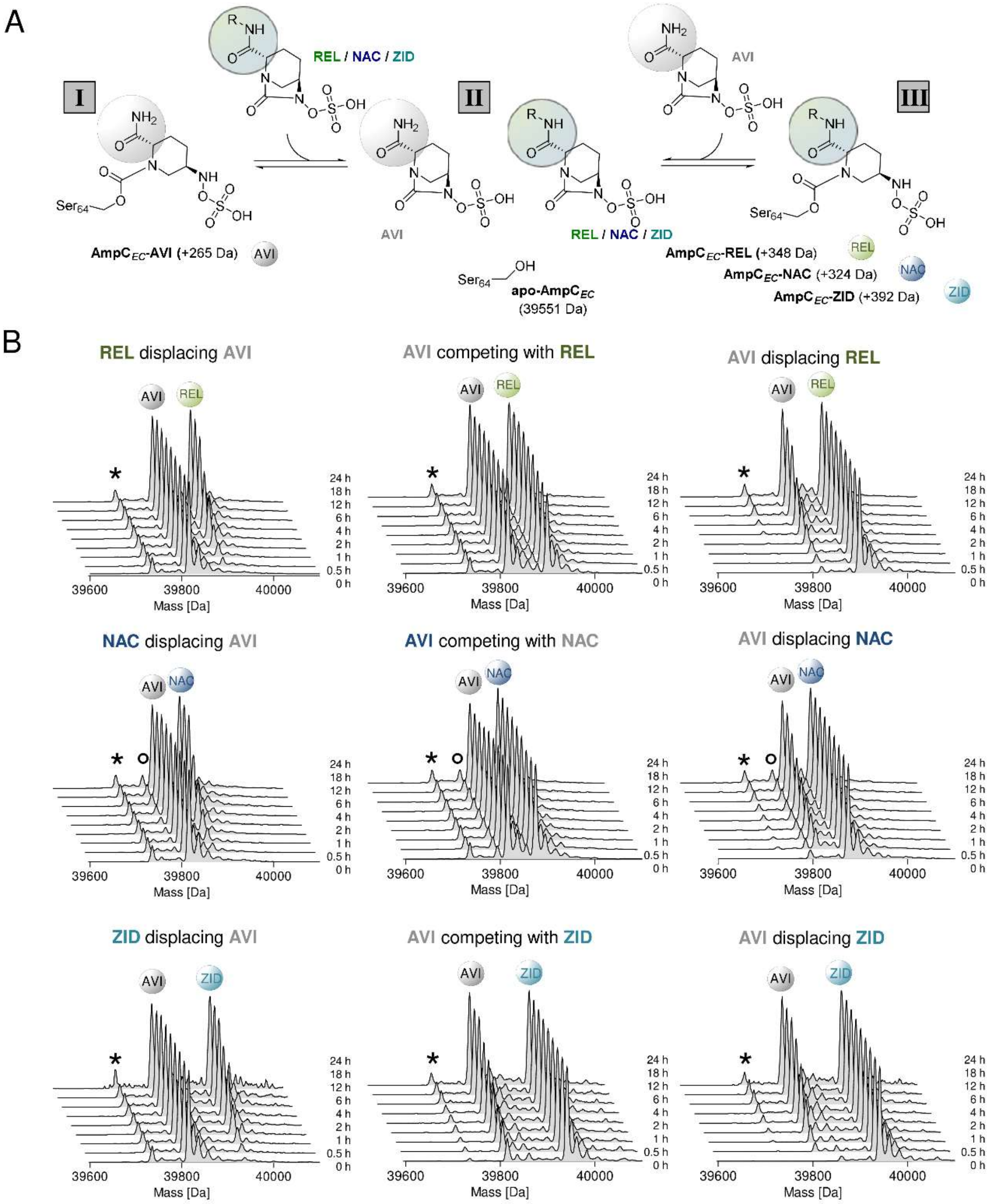

Figure S4: Mass spectrometric analyses investigating reversible DBO binding. (A) Schematic of intact protein SPE-MS observed DBO competition experiments. (B) SPE-MS analyses of competition exteriments. Left: AVI carbamoyl-enzyme displacement by REL/NAC/ZID (I). Center: Direct competition between AVI and REL/NAC/ZID (II). Right: REL/NAC/ZID carbamoyl-enzyme displacement by AVI (III). AmpC$_{EC}$ (3 μM) was incubated with 1.1 equiv. DBO. For direct competition experiments (center) both DBOs were added simultaneously; for displacement experiments (left and right) the second DBO was added after 5 min incubation of AmpC$_{EC}$ with the first DBO. Note, low abundancy signals with -80 Da mass decreases relative to the intact DBO complexes were observed, especially for AVI and NAC (labelled * and ∘, respectively). These are likely artefacts of the MS process (see text and Figure S6). Buffer: 50 mM Tris, pH 7.5. Deconvoluted spectra are shown, as derived using the maximum entropy algorithm in the MassHunter Workstation Qualitative Analysis V.7 programme (Agilent Technologies). Proposed assignments of the observed mass-shifts are given in Table S6.

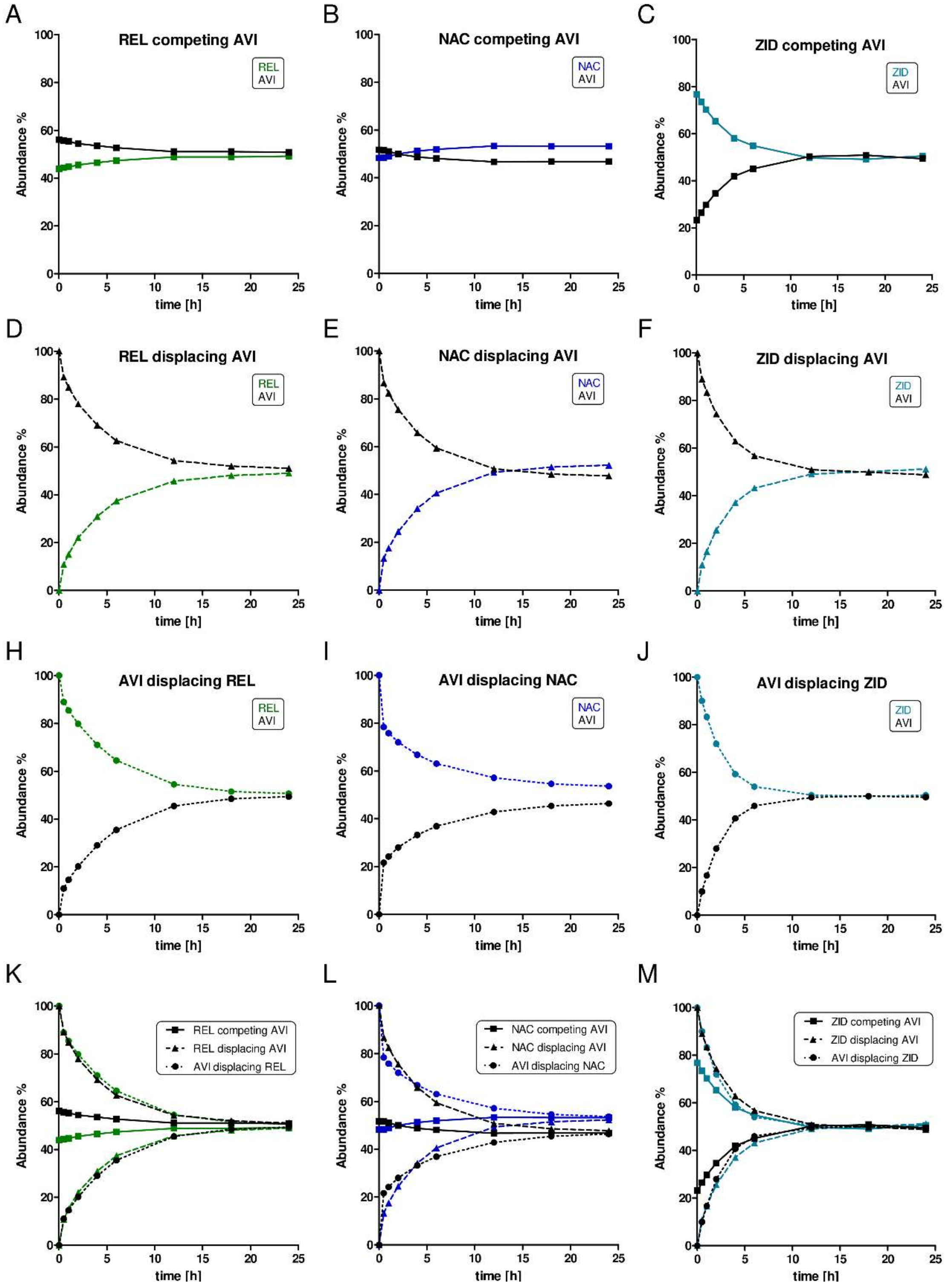

Figure S5: Binding to AmpC$_{EC}$ of AVI in competition with REL, NAC, or ZID as observed by SPE MS. AmpC$_{EC}$ (3 μM) was incubated with 1.1 equiv DBOs in 50 mM Tris, pH 7.5. Experiments were performed in duplicate. Reactions were monitored via protein observed SPE-MS. Spectra were deconvoluted (Figure S4) using the maximum entropy algorithm in the MassHunter Workstation Qualitative Analysis V.7 programme (Agilent Technologies). The relative peak areas (in % abundance) of the +265 Da, + 338 Da, +324 Da or +391 Da adducts for AVI, REL, NAC, and ZID are shown. Note, low abundancy signals with a -80 Da mass reduction relative to the intact DBO complexes were observed for all DBO carbamoyl-enzyme complexes, but with varying intensities for the different DBOs; the -80 Da mass reductions are likely artefacts of the MS process (see text and Figure S6).

Figure S5 (cont.): The peak areas were corrected for the intensities of the -80 Da species. (A-C) Direct Competition between AVI and REL/NAC/ZID. AmpC$_{EC}$ was added to a mixture of AVi (1.1 equiv.) and REL, NAC, or ZID (1.1 equiv.). (D-F) AVI acyl-enzyme displacement by REL/NAC/ZID. REL, NAC, or ZID (1.1 equiv.) were added to an AmpC$_{EC}$-AVI complex obtained via icubation of AmpC$_{EC}$ with AVI (1.1 equiv. for 10 min. (H-J) REL/NAC/ZID displacement by AVI (III shown in (A)). AVI (1.1 equiv.) was added to an AmpC$_{EC}$-REL, -NAC, or -ZID complex obtained via incubation of AmpC$_{EC}$ with REL, NAC, or ZID (1.1 equiv.) for 10 min. Note the top three panels in this figure are present in Figure 2; they are included here to enable comparison.

A

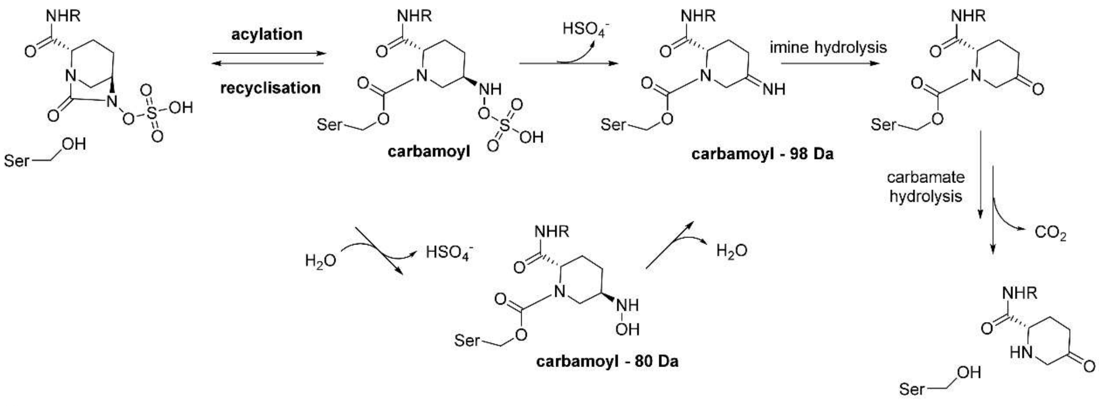

B

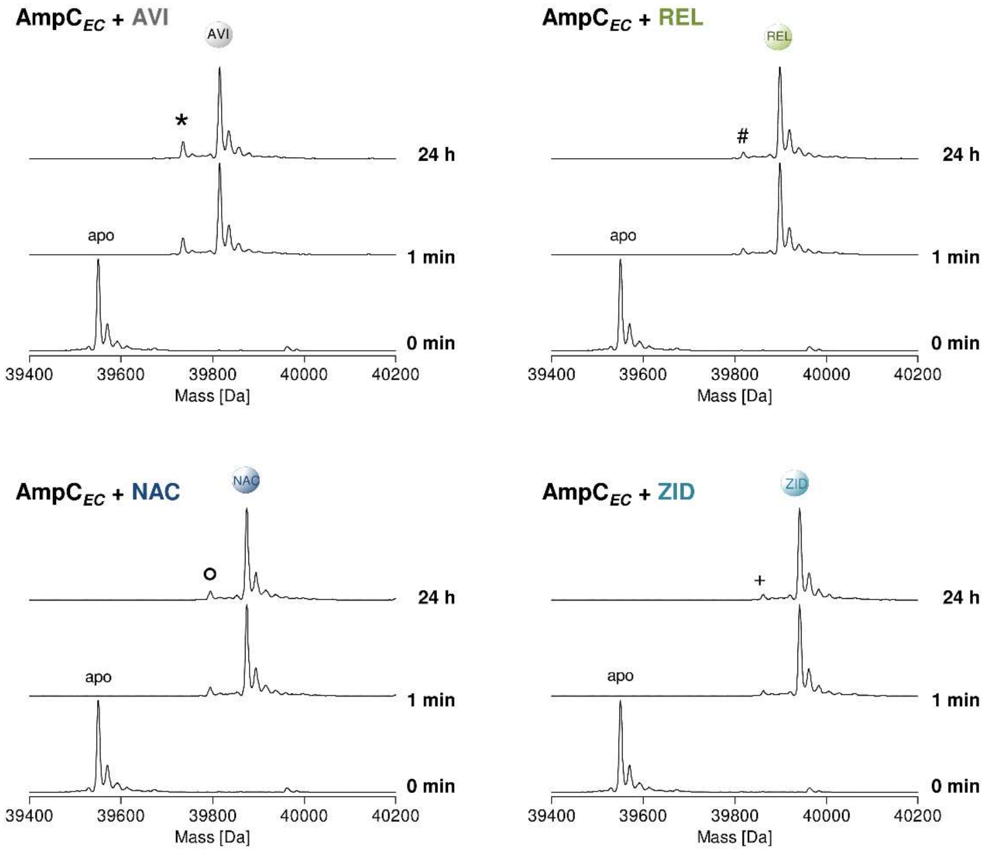

Figure S6: SPE-ESI MS time courses for AmpC*EC* modification by AVI, REL, NAC, or ZID. (A) Schematic for fragmentation of DBO derived complexes by the KPC β-lactamases; note that an analogous fragmentation can be induced under mass-spectrometry conditions. (B) AmpC*EC* (3 µM) was incubated with AVI, REL, NAC, or ZID (3.3 µM) and incubated at room temperature. Buffer: 50 mM Tris-HCl, pH 7.5. Mass-spectra were acquired both before and after incubation with the inhibitor for the indicated incubation times. Deconvoluted mass spectra are shown, as derived using the maximum likely entropy algorithm in the MassHunter Workstation Qualitative Analysis V.7 programme (Agilent Technologies). Proposed assignments of the observed mass-shifts are given in Table S6. Note, the intensities of carbamoyl -80 Da adducts (marked *,$^\#$,$^\circ$, and $^+$) remain constant relative to the +265 Da, +338 Da, +324 Da or +391 Da adducts for AVI, REL, NAC and ZID, respectivley (AVI 16 % abundancy, REL: 7 % abundancy, NAC 10 % abundancy, ZID: 6 % abundancy), and are therefore likely artefacts of the ionization process.

Table S6: Modifications of AmpC$_{EC}$ by AVI, REL, NAC, or ZID as analysed by SPE-MS. Differences between measured and calculated masses are within the experimental error. [a]Masses derived from deconvolution of measured spectra using the maximum likely entropy algorithm in the MassHunter Workstation Qualitative Analysis V.7 programme (Agilent Technologies). [b]Relative to the measured unmodified protein mass.

	Mass observed[a] [Da]	Mass calculated [Da]	Mass shift observed[b] [Da]	Mass shift calculated [Da]	Assignment
**AmpC$_{EC}$**	39551	39551			
+ AVI	39816	39816	+ 265	+ 265	AmpC$_{EC}$-AVI
	39736	39736	+ 185	+ 185	* carbamoyl – 80 Da (minor, potential artefact)
+ REL	39899	39899	+ 348	+ 348	AmpC$_{EC}$-REL
	39816	39819	+ 265	+ 268	#carbamoyl – 80 Da (minor, potential artefact)
+ NAC	39875	39875	+ 324	+ 324	AmpC$_{EC}$-NAC
	39796	39795	+ 245	+ 244	°carbamoyl – 80 Da (minor, potential artefact)
+ ZID	39942	39942	+ 391	+ 391	AmpC$_{EC}$-ZID
	39857	39862	+ 306	+ 311	+carbamoyl – 80 Da (minor, potential artefact)

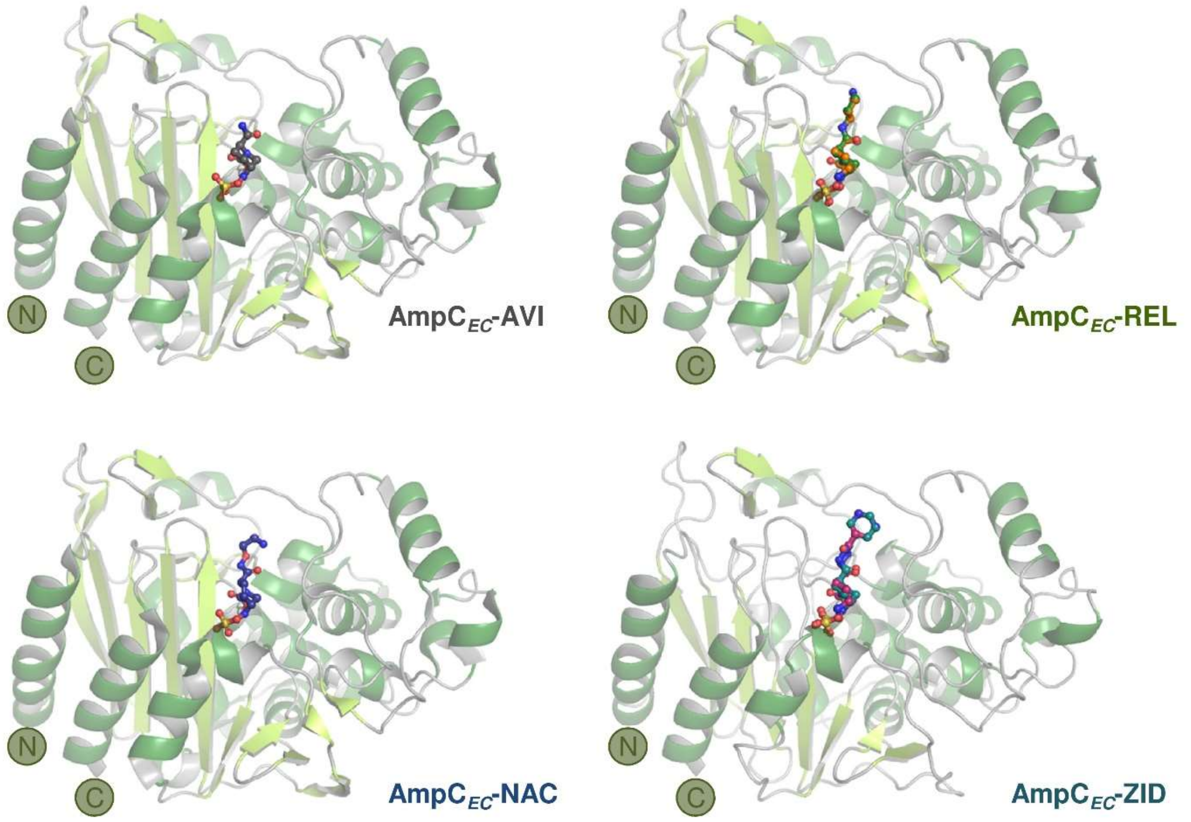

Figure S7: Overview of AmpC$_{EC}$–DBO complex structures. β-Strands are in pale green, α-helices are in dark green. C- and N-termini are marked C and N, respectivley. Bound DBOs are shown as sticks in grey (AVI), green (REL conformation A), orange (REL conformation B), blue (NAC), cyan (ZID conformation A), and pink (ZID conformation B). PDB IDs: 6TBW (AmpC$_{EC}$-ZID), 6TPM (AmpC$_{EC}$-REL), 6T7L (AmpC$_{EC}$-NAC), 6T5Y (AmpC$_{EC}$-ZID).

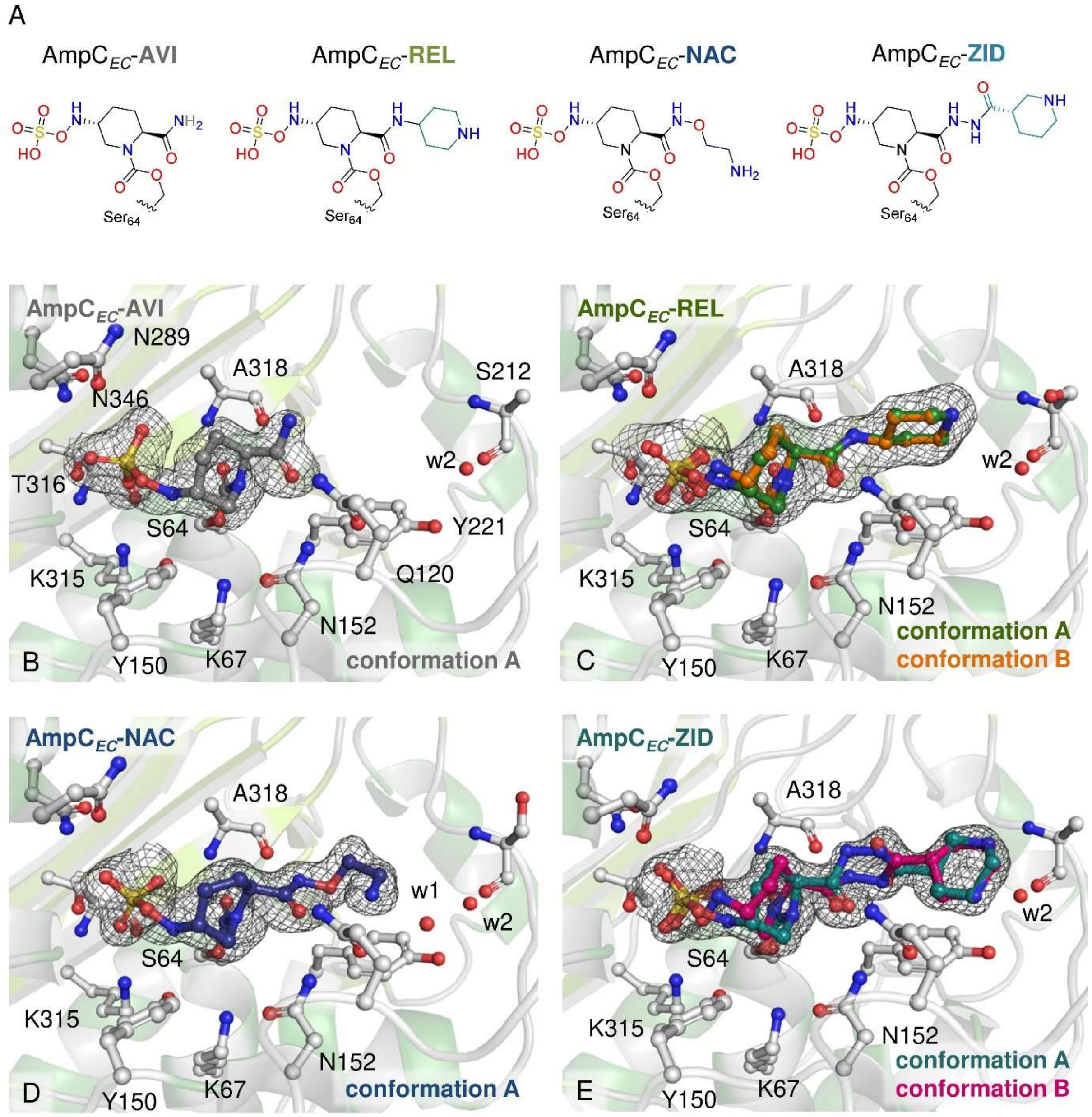

Figure S8: Views of active sites of AmpC_{EC}-DBO complexes. (A) Structures of DBOs in their ring opened forms, after reaction with the nucleophilic Ser. (B - E) Active site views of AVI, REL, NAC, and ZID bound to AmpC_{EC}. AVI and NAC are observed in a single conformation (A, note that the *N*-sulfate nitrogen is proximate to the phenolic OH of Y150). REL and ZID are both modelled in two conformations (A, and B), with the *N*-sulfate nitrogen being proximate to Y150 only in conformation A). In the case of ZID two conformations are observed for the acyl amino sidechain. Polder omit maps[3] are shown as grey mesh and contoured at 3.0 σ.

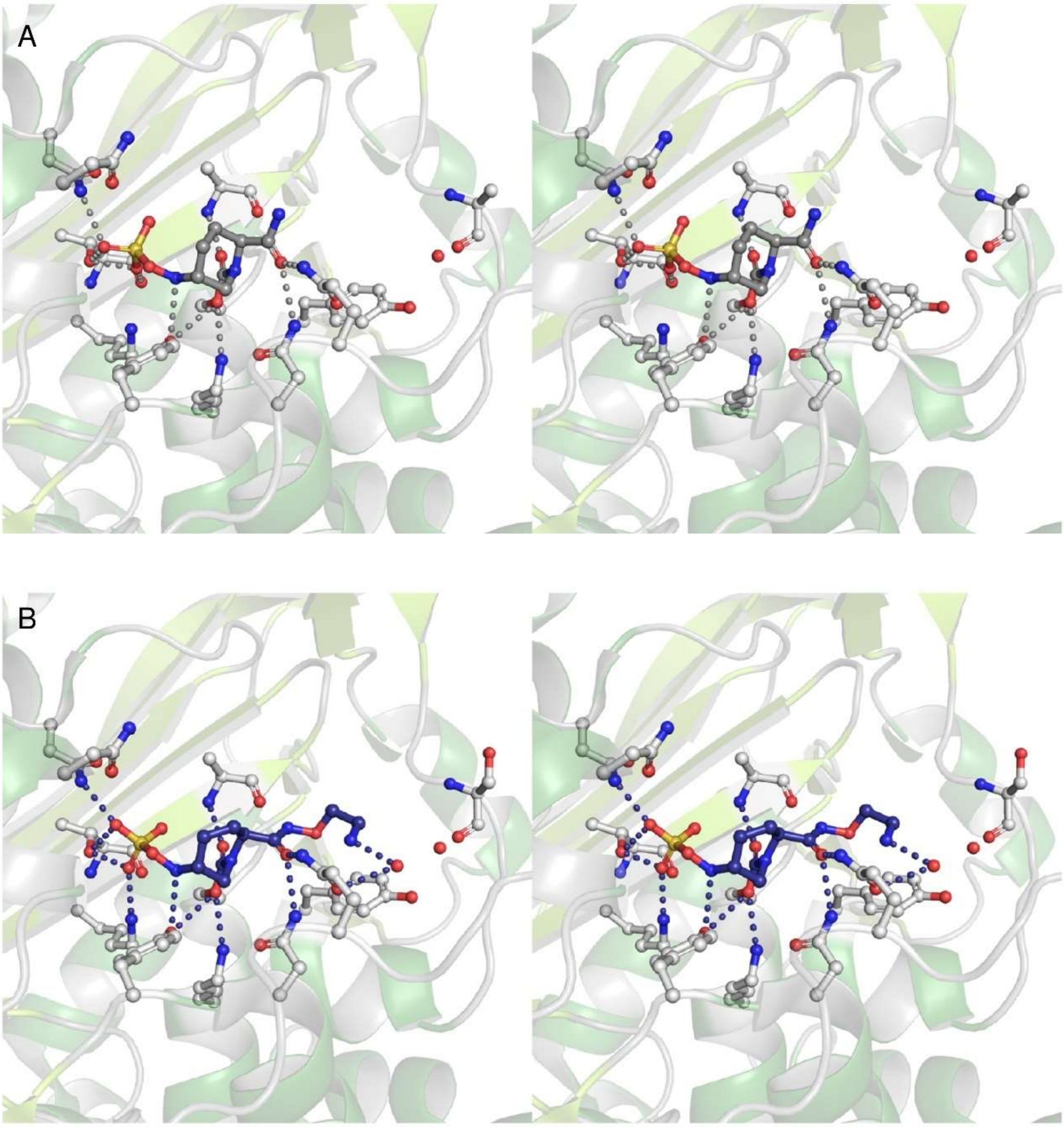

Figure S9: Stereo-views of AmpC$_{EC}$-AVI and AmpC$_{EC}$-NAC complexes. The figure shows convergent (cross-eye) stereo-views of the activesite interactions as shown in Figure 4A and B, a) Ampc$_{EC}$-AVI complex, PDB ID: 6TBW, b) Ampc$_{EC}$-NAC complex, PDB ID: 6T7L.

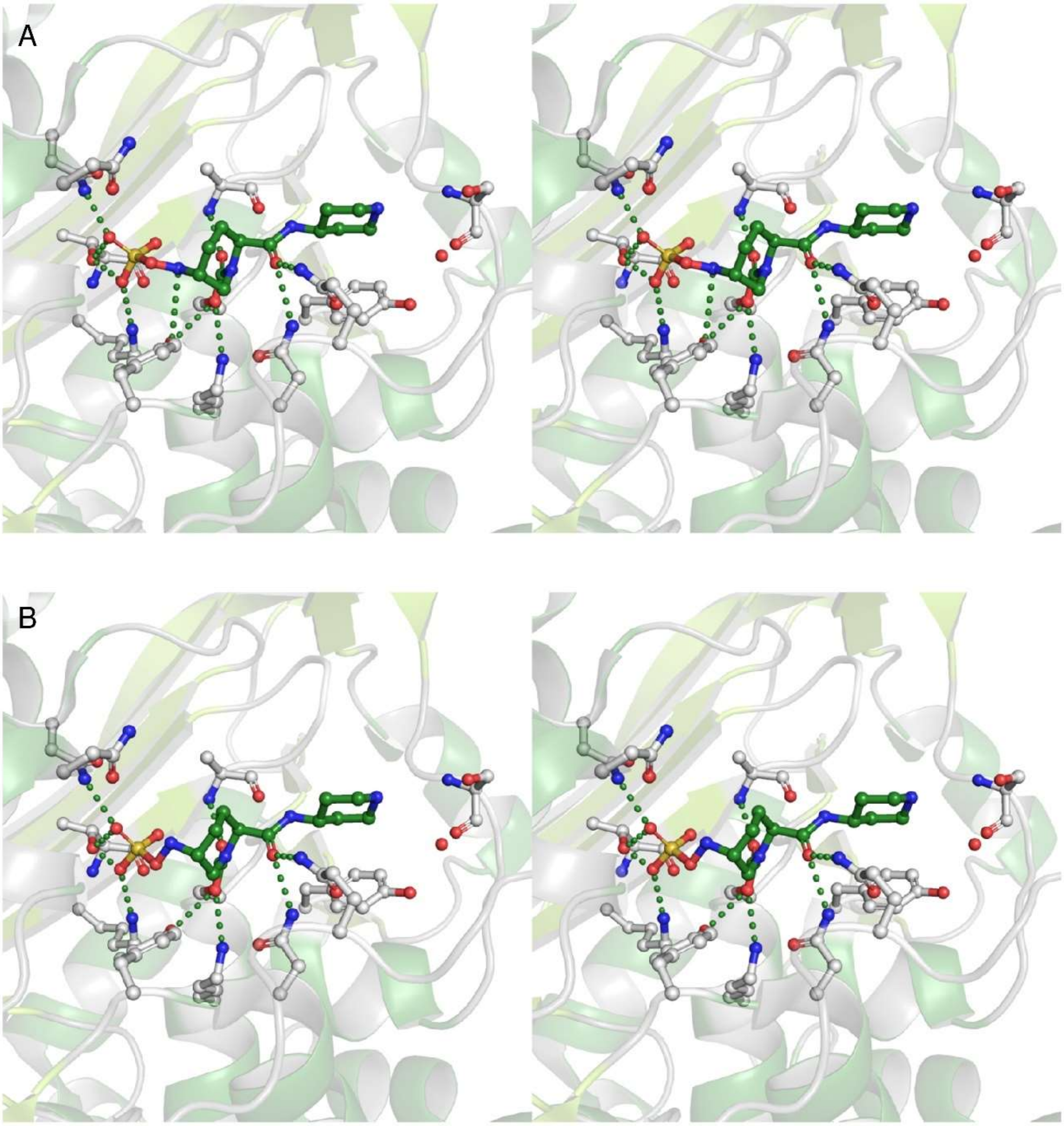

Figure S10: Stereo-views of AmpC$_{EC}$-REL complex. The figure shows convergent (cross-eye) stereo-views of the activesite interactions as shown in Figure 4C and D, a) REL in conformation A, b) REL in conformation B. PDB ID: 6TPM.

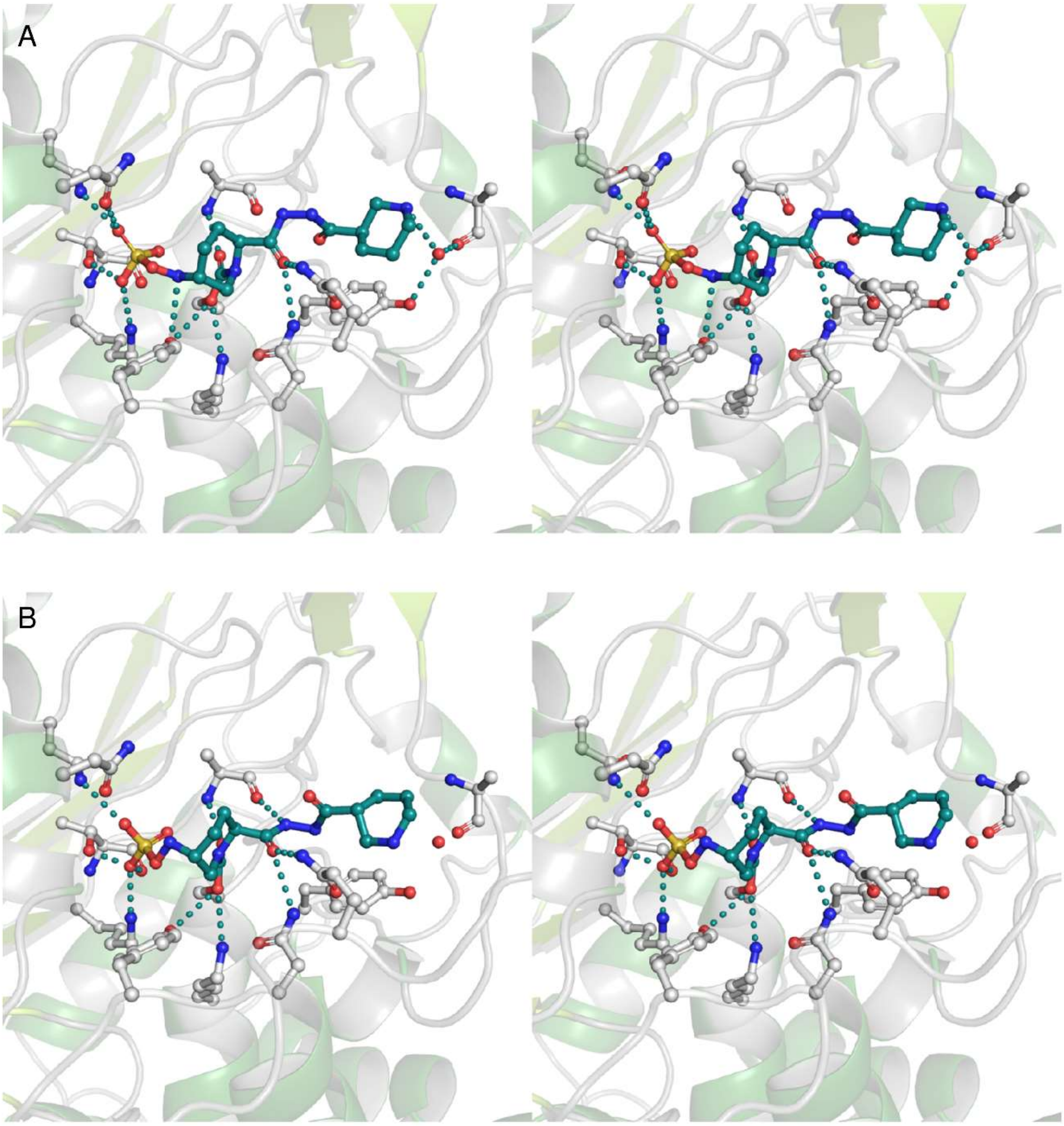

Figure S11: Stereo-views of AmpC$_{EC}$-ZID complex. The figure shows convergent (cross-eye) stereo-views of the activesite interactions as shown in Figure 4D and E, a) ZID in conformation A, b) ZID in conformation B. PDB ID: 6T5Y.

Table S7: Distances for polar interactions of AVI, REL, NAC, and ZID in their ring opened forms with AmpC$_{EC}$ active site residues.

		Distances to ring opened diazabicyclooctane (Å) (the observed *N*-sulfate conformation A or B is given in parentheses)					
**AmpC$_{EC}$ atom**	**DBO atom**	**AVI (A)**	**REL (A)**	**REL (B)**	**NAC (A)**	**ZID (A)**	**ZID (B)**
Ala318, backbone N	DBO carbonyl O	2.77	2.79	2.83	2.94	2.83	2.82
Thr316, sidechain O	SO_4 O1	2.62	2.54	2.30	2.55	2.74	2.46
Thr316, backbone O	SO_4 O1	2.94	-	-	-	-	2.90
Lys315, sidechain N	SO_4 O1	-	3.00	2.91	2.91	3.09	2.79
Asn346, sidechain N	SO_4 O2	2.98	2.60	2.92	2.70	2.64	3.03
Thr316, sidechain O	SO_4 O2	-	2.95	-	3.09	-	-
Asn152, sidechain N	amide O	3.08	2.95	3.07	3.00	3.08	3.16
Gln120, sidechain N	amide O	2.90	2.89	2.78	3.06	3.12	3.03

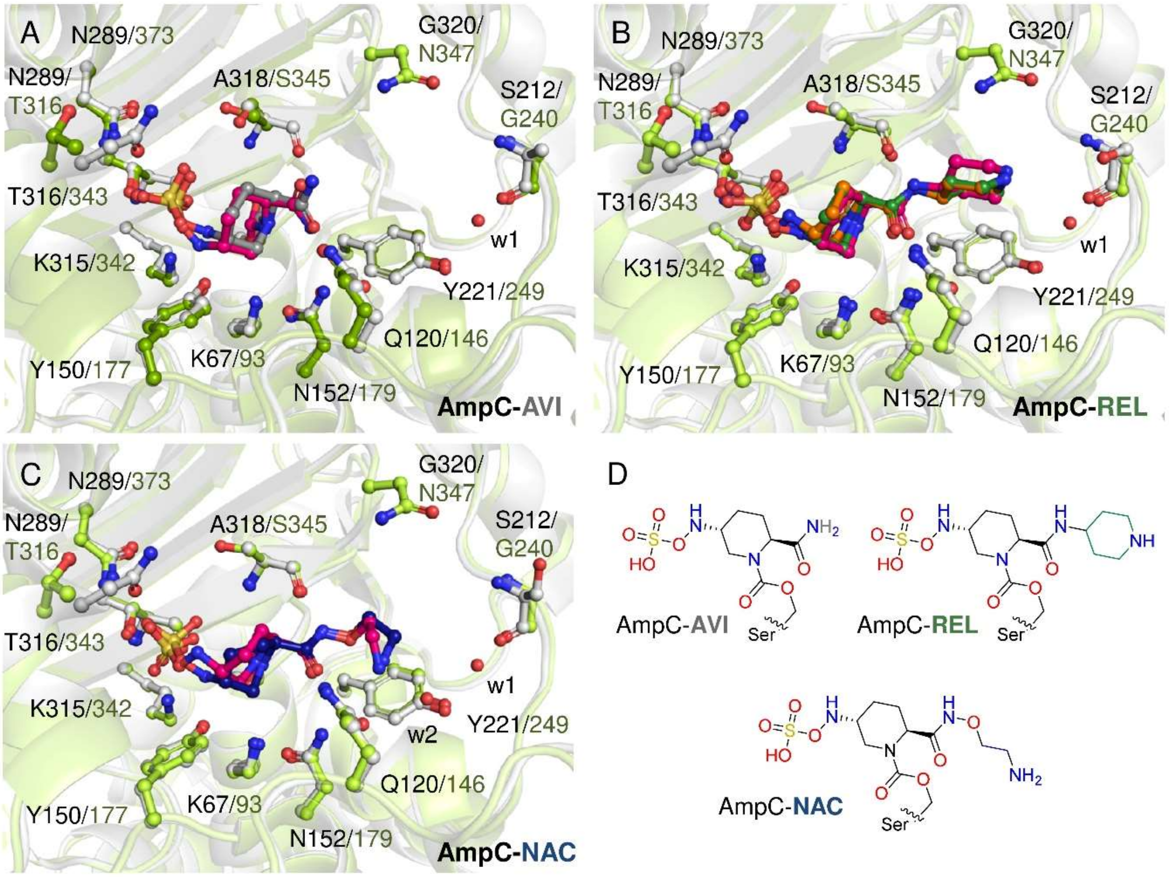

Figure S12: Overlay of AmpC$_{EC}$ structures with reported PDC-1 structures. (A) Overlay of the AmpC$_{EC}$-AVI complex structure (white/grey, PDB ID 6TBW) with the structure of the PDC-1-AVI complex[4] (green/pink, PDB ID 4OOY). (B) Overlay of the AmpC$_{EC}$ REL-acyl enzyme complex structure (white/green(conformation A)/organge (conformation B), PDB ID 6TPM) with that of the PDC-1-REL complex[4] (green/pink, PDB ID 4NK3). (C) Overlay of the AmpC$_{EC}$-NAC complex structure (white/darkblue, PDB ID 6T7L) with that of the PDC-1 NAC-acyl enzyme complex[5] (green/pink, PDB ID 4X68). Waters are shown as red spheres. (D) Structures of DBOs in their ring opened forms after reaction with an SBL.

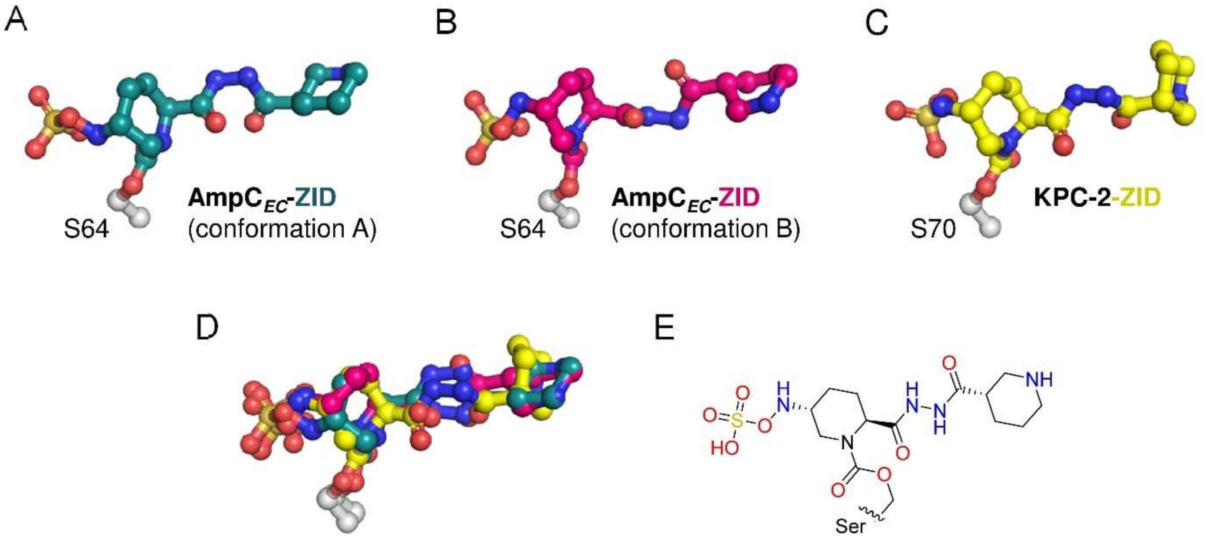

Figure S13: Comparison of ZID binding modes as observed in complex with AmpC$_{EC}$ and KPC-2. (A) Conformation A of the ZID binding mode at the AmpC$_{EC}$ active site. (B) Conformation B of the ZID binding mode at the AmpC$_{EC}$ active site. (C) The ZID binding mode at the KPC-2 active site (PDB ID: 6B1J) [2]. (D) Overlay of ZID binding modes, color-coded as in (A-C). (E) Structure of ring-opened ZID bound to the nucleophlic serine.

References

1. van Berkel, S.S.; Brem, J.; Rydzik, A.M.; Salimraj, R.; Cain, R.; Verma, A.; Owens, R.J.; Fishwick, C.W.G.; Spencer, J.; Schofield, C.J. Assay platform for clinically relevant metallo-β-lactamases. *J. Med. Chem.* **2013**, *56*, 6945-6953.
2. Papp-Wallace, K.M.; Nguyen, N.Q.; Jacobs, M.R.; Bethel, C.R.; Barnes, M.D.; Kumar, V.; Bajaksouzian, S.; Rudin, S.D.; Rather, P.N.; Bhavsar, S., et al. Strategic Approaches to Overcome Resistance against Gram-Negative Pathogens Using β-Lactamase Inhibitors and β-Lactam Enhancers: Activity of Three Novel Diazabicyclooctanes WCK 5153, Zidebactam (WCK 5107), and WCK 4234. *J. Med. Chem.* **2018**, *61*, 4067-4086.
3. Liebschner, D.; Afonine, P.V.; Moriarty, N.W.; Poon, B.K.; Sobolev, O.V.; Terwilliger, T.C.; Adams, P.D. Polder maps: improving OMIT maps by excluding bulk solvent. *Acta Crystallogr. Sect. D. Biol. Crystallogr.* **2017**, *73*, 148-157.
4. Lahiri, S.D.; Johnstone, M.R.; Ross, P.L.; McLaughlin, R.E.; Olivier, N.B.; Alm, R.A. Avibactam and class C β-lactamases: mechanism of inhibition, conservation of the binding pocket, and implications for resistance. *Antimicrob. Agents Chemother.* **2014**, *58*, 5704-5713.
5. Morinaka, A.; Tsutsumi, Y.; Yamada, M.; Suzuki, K.; Watanabe, T.; Abe, T.; Furuuchi, T.; Inamura, S.; Sakamaki, Y.; Mitsuhashi, N., et al. OP0595, a new diazabicyclooctane: mode of action as a serine β-lactamase inhibitor, antibiotic and β-lactam 'enhancer'. *J. Antimicrob. Chemother.* **2015**, *70*, 2779-2786.
